# Supplementary material for: Axonemal structures reveal mechanoregulatory and disease mechanisms
Source: Nature. 2023 May 31;618(7965):625–33. doi: 10.1038/s41586-023-06140-2 (PMC10266980; doi:10.1038/s41586-023-06140-2)
Supplement: Supplementary file 1 — Supplementary Information [file 41586_2023_6140_MOESM1_ESM.pdf]

nature portfolio

https://doi.org/10.1038/s41586-023-06140-2

Supplementary information

Axonemal structures reveal mechanoregulatory and disease mechanisms

In the format provided by the authors and unedited

SI Guide

Axonemal structures reveal mechanoregulatory and disease mechanisms

Travis Walton, Miao Gui, Simona Velkova, Mahmoud R. Fassad, Robert A. Hirst, Eric Haarman, Christopher O'Callaghan, Mathieu Bottier, Thomas Burgoyne, Hannah M. Mitchison, Alan Brown

This file contains:

Section 1. Supplementary Tables.

SI Table 1 Proteins identified within the 96-nm modular repeat of the *C. reinhardtii* doublet microtubule..........2
SI Table 2 Rationale for the placement of *C. reinhardtii* axonemal proteins.........5
SI Table 3 External proteins identified within the 96-nm modular repeat of the human doublet microtubule......9
SI Table 4 Rationale for the placement of human axonemal proteins..........11
SI Table 5 Structure-verified axonemal proteins associated with disease..........13

Section 2. Figures related to cryo-EM processing of the *C. reinhardtii* axoneme.

SI Fig. 1 Overview of the processing strategy used to generate a composite map and atomic model of the *C. reinhardtii* doublet microtubule (DMT)..........16
SI Fig. 2 Processing strategy for the 96-nm modular repeat..........18
SI Fig. 3 Processing strategy for outer dynein arms (ODA)..........19
SI Fig. 4 Processing strategy for the nexin-dynein regulatory complex (N-DRC)..........20
SI Fig. 5 Processing strategy for radial spoke 3 (RS3S) and the bases of IDA*g*/*d*..........21
SI Fig. 6 Processing strategy for the distal protrusion (DP)..........22
SI Fig. 7 Processing strategy for the N-DRC linker..........23
SI Fig. 8 Processing strategy for the inner dynein arm *f* (IDA*f*) core..........24
SI Fig. 9 Processing strategy for the tether/tetherhead (T/TH) complex and associated IDA*f* motors..........25

Section 3. Figures related to model building of the *C. reinhardtii* axoneme.

SI Fig. 10 AI-enabled modeling of IDA*f*..........26
SI Fig. 11 AI-enabled modeling of the IDA*f* tether/tetherhead (T/TH) complex..........28
SI Fig. 12 AI-enabled modeling of the N-DRC..........29

Section 4. Figures related to cryo-EM processing of the human axoneme.

SI Fig. 13 Processing of the human doublet microtubule (DMT)..........31
SI Fig. 14 Cryo-EM maps and Fourier Shell Correlation (FSC) curves for human axonemal complexes..........32

Section 5. SI video legends.

SI Video 1 Rotation of the *C. reinhardtii* doublet microtubule..........34
SI Video 2 Coordinated movement of IDA*f* with ODA1 and 2..........34
SI Video 3 Coordinated movement of N-DRC with ODA3..........34
SI Video 3 High-speed video microscopy of human respiratory cilia..........34

Section 1. Supplementary Tables

Supplementary Table 1 | Proteins identified within the 96-nm modular repeat of the *C. reinhardtii* doublet microtubule. The table excludes microtubule inner proteins (MIPs) and components of radial spokes 1 and 2 if they have been reported elsewhere[13,14]. Abbreviations: CaM, calmodulin; FAP, flagellar associated protein; DP, distal protrusion; IDA*f*, inner dynein arm *f*; MIA, modifier of inner arms; ODA, outer dynein arm; ODA-DC, ODA docking complex; RS3S, radial spoke 3 stand-in; T/TH, tether/tetherhead; LC, light chain; IC, intermediate chain; HC, heavy chain.

Complex	Protein	Phytozome ID	Length (residues)	Molecular weight (kDa)	Modeled residues
**MIP**	FAP1	Cre02.g144450.t1.2<br>A8J0T8 (Uniprot)	196	21.7	4-196
	FAP77	A8IB22 (Uniprot)	270	29.2	15-243
	FAP144	Cre02.g095072.t1.1<br>A8JG37 (Uniprot)	144	16.2	9-103, 109-132
	FAP257	Cre17.g724400.t1.2<br>A8J6L0 (Uniprot)	286	32.0	54-234
**External coiled coils**	CCDC39	Cre17.g701250.t1.2<br>A0A0A1H1F6 (Uniprot)	925	105.3	33-729
	CCDC40	Cre17.g698365_4532.1	904	101.2	24-727
	FAP184 (CCDC96)	Cre03.g160050.t1.2	490	55.1	151-321, 325-418, 423-474
	FAP263 (CCDC113)	Cre17.g717150.t1.2	409	45.3	19-220, 226-321, 327-375
	FAP299	Cre11.g482200.t1.2<br>A0A2K3D8T3 (Uniprot)	237	27.4	1-237
	FAP57	Cre04.g217914.t1.1<br>A0A2K3DTF4 (Uniprot)	1316	146.3	1-771, 810-1050, 1060-1123
	FAP189	Cre13.g584400.t1.2<br>A8HUA7 (Uniprot)	868	102.0	289-722, 727-768, 777-836
**ODA**	α-HC	Cre03.g145127.t1.1	4503	504.4	1-648, 742-4500
	β-HC	Cre09.g403800.t1.2	4568	519.9	18-699, 731-4568
	γ-HC	UniProt: Q39575	4485	512.8	1-4485
	IC1	Cre12.g536550.t1.2	682	76.3	47-221, 248-457, 474-675
	IC2	Cre12.g506000.t1.2	567	63.5	1-517, 535-550
	LC1	Cre02.g092850.t1.2<br>Q9XHH2 (Uniprot)	198	22.2	2-196
	LC2	Cre12.g527750.t1.2<br>O04355 (Uniprot)	136	15.9	12-135
	LC3	Cre12.g528850.t1.1<br>Q39592 (Uniprot)	156	17.4	1-156
	LC4	Cre01.g051250.t1.2<br>Q39584 (Uniprot)	159	17.8	15-90, 97-158
	LC5	Cre17.g714250.t1.2<br>Q39591 (Uniprot)	129	14.2	1-129
	LC6	Cre03.g187200.t1.2<br>Q39579 (Uniprot)	120	13.9	14-120
	LC7a	Cre08.g376550.t1.2	105	11.9	2-104
	LC7b	Cre12.g546400.t1.2<br>A8IY95 (Uniprot)	100	11.1	2-95
	LC8	Cre03.g181150.t1.1	91	10.3	7-91

		Q39580 (Uniprot)			
	LC9	Cre10.g428850.t1.2<br>Q2VIY5 (Uniprot)	117	13.0	1-117
	LC10	Cre12.g527800.t1.1<br>A8J5C4 (Uniprot)	103	12.1	5-102
**ODA-DC**	DC1	Cre17.g703850.t1.2<br>A8IPZ5 (Uniprot)	749	83.4	84-403, 411-460, 568-601
	DC2	Cre16.g666150.t1.2<br>A8JF70 (Uniprot)	552	62.2	30-61, 67-247, 295-465, 476-494
	DC3	Cre14.g617550.t1.2<br>Q7Y0H2 (Uniprot)	184	21.3	4-173
**IDA*f***	IDA*f* α-HC	Cre12.g484250.t1.1	4625	522.8	1-53, 178-4624
	IDA*f* β-HC	Cre14.g624950.t1.1	4513	510.7	12-4511
	IC97	Cre14.g631200.t1.2	760	81.6	46-168, 207-<br>261, 306-760
	IC138	Cre12.g520950.t1.1<br>Q5XPS1 (Uniprot)	1057	111.2	195-265, 356-377, 412-450, 581-639, 709-1057
	IC140	Cre16.g674515.t1.1<br>A8J3B6 (Uniprot)	1024	109.8	283-437, 448-1024
	FAP120	Cre02.g116400.t1.1<br>A8I2N3 (Uniprot)	294	31861.12	25-207
	LC7a	Cre08.g376550.t1.2	105	11.9	1-105
	LC7b	Cre12.g546400.t1.2<br>A8IY95 (Uniprot)	100	11.1	1-100
	LC8	Cre03.g181150.t1.1<br>Q39580 (Uniprot)	91	10.3	1-91
	Tctex1	Cre01.g004250.t1.2<br>O64980 (Uniprot)	114	12.7	1-114
	Tctex2b	Cre09.g394213.t1.1<br>Q6IET4 (Uniprot)	120	13.7	1-120
**MIA**	FAP73	Cre16.g689600.t1.2<br>M1V4Y8 (Uniprot)	308	35.6	36-308
	FAP100	Cre02.g103950.t1.2<br>A8I4E9 (Uniprot)	576	65.2	121-530
**T/TH**	FAP43	Cre16.g691440.t1.1	1950	199.4	1-815, 872-1202, 1259-1441, 1612-1777, 1800-1915
	FAP44	Cre09.g386736.t1.1	2141	221.9	392-951, 1027-1159, 1201-1796, 1848-2006, 2025-<br>2080, 2099-2136
	MOT7	Cre01.g038750.t1.2<br>A0A2K3E783 (Uniprot)	238	26.2	1-238
**Single-<br>headed IDAs**	IDA*a* (DHC6)	Cre05.g244250.t1.2	4309	477.7	205-550, 590-636, 685-4307
	IDA*b* (DHC5)	Cre02.g107050.t1.1	4185	463.9	113-339, 365-493, 560-1990, 2035-4183
	IDA*c* (DHC9)	Cre02.g141606.t1.1	4151	464.6	50-80, 216-545, 563-4150
	IDA*d* (DHC2)	Cre09.g392282.t1.1	4165	467.0	65-323, 376-4165
	IDA*e* (DHC8)	Cre16.g685450.t1.1	4209	470.2	151-364, 406-4209
	IDA*g* (DHC7)	Cre14.g627576.t1.1	4191	468.3	144-466, 518-656, 700-4189
	Actin (IDA5)	Cre13.g603700.t1.2<br>A8JAV1 (Uniprot)	377	41.8	1-377
	Centrin	Cre11.g468450.t1.2<br>A8JC40 (Uniprot)	169	19.5	1-169
	p28	Q39604 (Uniprot)	253	28.7	1-21, 46-240
	p38	Cre03.g186300.t1.2<br>A4PET3 (Uniprot)	380	40.9	1-380
	p44	Cre08.g374650.t1.1	423	46.1	1-20, 48-423

**RS3S**	FAP61	Cre03.g167600.t1.2<br>A8IF44 (Uniprot)	1147	121.3	1-302, 351-1147
	FAP91	Cre07.g330700.t1.2	1298	134.3	181-200, 213-234, 244-398, 414-440, 535-659
	FAP251	Cre03.g185650.t1.2	906	97.4	1-906
**N-DRC**	DRC1	P0DL09 (Uniprot)	698	79.3	47-413, 455-588, 615-698
	DRC2	Cre13.g607750.t1.2<br>A8JB22 (Uniprot)	573	64.9	1-374, 448-533
	DRC3	Cre12.g531500.t1.2<br>A8IVX2 (Uniprot)	524	60.4	1-524
	DRC4	Cre11.g476850.t1.2<br>Q7XJ96 (Uniprot)	471	55.1	1-466
	DRC5	Cre01.g045350.t1.2	390	43.5	1-390
	DRC6	Cre12.g515200.t1.2<br>A8JHD7 (Uniprot)	260	28.5	1-208
	DRC7	Cre14.g612700.t1.2	1598	177.5	1-610, 622-809, 840-902, 924-1006, 1139-1441, 1491-1598
	DRC8	Cre16.g676645.t1.1<br>A8J3A0 (Uniprot)	166	18.6	1-166
	DRC9	Cre01.g025450.t1.2	406	46.4	1-406
	DRC10	Cre02.g141350.t1.2<br>A8J0N6 (Uniprot)	356	40.9	1-356
	DRC11	Cre07.g325762.t1.1	836	94.7	1-836
	CaM	Cre03.g178150.t1.1	163	18.3	1-163
**DP**	FAP78	Cre12.g536600.t1.1<br>A0A2K3D574 (Uniprot)	2520	267.1	148-256, 297-715, 925-1005, 1033-1130, 1154-1231, 1297-1352, 1363-1411

Supplementary Table 2 | Rationale for the placement of *C. reinhardtii* axonemal proteins. Fitting of atomic models within the cryo-EM map of the 96-nm modular repeat of the *C. reinhardtii* axoneme often involved incorporating information from multiple sources. A non-exhaustive list of information sources included: 1) sidechain density features which enabled *de novo* model building, 2) consistency of density with known or predicted atomic models, 3) analysis of subtomogram averages of *C. reinhardtii* axonemes from mutant strains lacking individual proteins or axonemal complexes, 4) analysis of subtomogram averages of *C. reinhardtii* axonemes from strains expressing tagged proteins, and 5) consistency of models with predictions of protein-protein interactions. Only proteins newly positioned within this study are shown. Other microtubule inner proteins (MIPs), ODA and radial spoke components are provided in previous publications[5,13,14]. Abbreviations: FAP, flagellar associated protein; DP, distal protrusion; IDA*f*, inner dynein arm *f*; MIA, modifier of inner arms; ODA-DC, ODA docking complex; OID, outer-inner dynein link; RS3S, radial spoke 3 stand-in; T/TH, tether/tetherhead; LC, light chain; IC, intermediate chain; HC, heavy chain.

Complex	Protein	ID	Evidence for location
MIP	FAP1	Cre02.g144450.t1.2	1) Well-resolved sidechain density agrees with the expected primary sequence
	FAP77	Cre10.g432850.t1.2	1) The location is consistent with the position of CFAP77 in the human respiratory DMT[64] and bovine sperm DMT[85]<br>2) Well-resolved sidechain density agrees with the expected primary sequence
	FAP144	Cre02.g095072.t1.1	1) The location is consistent with the position of the homologous FAM183A/CFAP144 in the human DMT[64]
	FAP257	Cre17.g724400.t1.2	1) Sidechain density agrees with the expected primary sequence
ODA	LC3	Cre12.g528850.t1.1	1) LC3 copurifies with ODA β-HC[86]<br>2) β-HC motor domain is not required for binding of LC3 to ODA[87]<br>3) The location is consistent with the position of *Tetrahymena* LC3 orthologs[4,6,88]<br>4) AlphaFold2 model of LC3 bound to helical bundles of β-HC matches our cryo-EM maps of ODA
	LC5	Cre17.g714250.t1.2	1) LC5 copurifies with ODA α-HC[86]<br>2) A *Chlamydomonas* strain that lacks α-HC (*oda11*) has reduced levels of LC5[89]<br>3) LC5 can be cross-linked to α-HC[90]<br>4) AlphaFold2 model of LC5 bound to helical bundles of α-HC is consistent with our cryo-EM maps of ODA
IDA*f*	$f\alpha$<br><br>$f\beta$	Cre12.g484250.t1.1<br><br>Cre14.g624950.t1.1	1) *ida1* and *ida2* mutants that lack the $f\alpha$ or $f\beta$ heavy chains do not assemble IDA*f* on axonemes[91]<br>2) AlphaFold2 model of N-terminal $f\alpha$/$f\beta$ dimerization domain places $f\alpha$ in orthologous position to ODA γ-HC, and $f\beta$ in orthologous position to ODA β-HC<br>3) Our cryo-EM maps position this model with $f\alpha$ closer to the IDA*b*, and $f\beta$ closer to the ODA array. These positions are consistent with the locations of the $f\alpha$ and $f\beta$ motor domains determined by partial rescue of *ida1* and *ida2* with N-terminal gene fragments and EM[92,93]
	IC97	Cre14.g631200.t1.2	1) IC97, IC138, FAP120 and LC7b are lost in *bop5* mutant axonemes[94]<br>2) Levels of IC97 are also reduced in *tctex2b* axonemes[95]<br>3) Cryo-ET of *bop5* and *tctex2b* mutant axonemes localizes IC97 to the proximal bottom and central regions of the IC-LC complex[67,95]<br>4) AlphaFold2 model predicts a distinctive fold for IC97 that fits our cryo-EM maps of IDA*f* in the lost region of *tctex2b* and *bop5* mutants<br>5) IC97 is not shared with other dyneins, and localizes outside the conserved core of multi-headed dyneins
	IC138	Cre12.g520950.t1.1	1) Forms a subcomplex with IC97, FAP120 and LC7b[94]<br>2) Encodes an N-terminal β-propeller and C-terminal tail with sequence conservation to ODA subunit IC1[96]<br>3) Cryo-ET of *bop5* mutants localizes the IC138-subcomplex near the connection between IDA*f* and IDA*b*[67]<br>4) Our cryo-EM maps of this region show a β-propeller surrounded by helical bundles, which matches our AlphaFold2 model of a IC138 subcomplex with $f\alpha$<br>5) This position is orthologous to ODA subunit IC1
	IC140	Cre16.g674515.t1.1	1) *ida7* mutants that lack IC140 do not assemble IDA*f* on their axonemes, suggesting a core location[97]<br>2) Encodes an N-terminal β-propeller and C-terminal tail with sequence conservation to ODA subunit IC2[98]<br>3) Cryo-ET of *ida7* mutant axonemes rescued with SNAP-IC140 localizes the N-terminus of IC140 at the proximal region of the ICLC structure[95]

			4) Our cryo-EM maps show a β-propeller, surrounded by the helical bundles of *f*β. An AlphaFold2 model of IC140 with *f*β matches this density.<br>5) This position is orthologous to ODA subunit IC2
	FAP120	Cre02.g116400.t1.1	1) Absent from *C. reinhardtii* mutants lacking IDA*f*[20]<br>2) FAP120 is a component of the IC138 subcomplex[20,94]<br>3) FAP120 does not coelute with IDA*f* by ion-exchange chromatography, suggesting a peripheral location [20]<br>4) Formed from ankyrin repeats, which generate a linear solenoid structure consistent with a density on the periphery of the IDA*f* core within the IC138 complex
	LC7a<br><br>LC7b	Cre08.g376550.t1.2<br><br>Cre12.g546400.t1.2	1) LC7a and LC7b copurify with IDA*f*[99]<br>2) LC7a is required for association of LC7b with IDA*f*, suggesting an interaction[99]<br>3) LC7b is part of the IC138 subcomplex, and cross-links with IC138[94,96,99]<br>4) Our cryo-EM maps show density for LC7a/b near IC138, which matches an AlphaFold2 model of LC7a/b bound to the tails of IC138 and IC140<br>5) Position is orthologous to a LC7a/b heterodimer in the ODA core[5]
	LC8	Cre03.g181150.t1.1	1) Multiple copies of LC8 are present in IDA*f*[100]<br>2) Our cryo-EM maps of IDA*f* show density for three LC8 dimers on top of the LC7a/b heterodimer<br>3) Three stacked dimers resemble the arrangement of LC8 in cytoplasmic dynein-2[101], *C. reinhardtii* ODA[5], and radial spokes[14]
	Tctex1<br><br>Tctex2b	Cre01.g004250.t1.2<br><br>Cre09.g394213.t1.1	1) Tctex1 co-purifies with IDA*f* and is missing in *ida1* mutant axonemes[100]<br>2) Tctex2b co-purifies with IDA*f* , and is highly reduced in *ida1* mutants[102]<br>3) Tctex1 levels are reduced in *tctex2b* mutant axonemes, suggesting an interaction[95]<br>4) Tctex2b localizes to structural defect observed in *tctex2b* mutant axonemes by cryo-ET[95]<br>5) Position is orthologous to the Tctex heterodimer in the ODA[5]
Tether/ tether-head (T/TH)	FAP43<br><br>FAP44	Cre16.g691440.t1.1<br><br>Cre09.g386736.t1.1	1) C-terminal coiled coil of FAP43/FAP44 identified based on sidechain densities near the inner junction<br>2) Identical location observed by cryo-ET for FAP43/44 C-termini labeled with strep-Au[25]<br>3) FAP43 requires FAP44 for its incorporation into the axoneme[24,25]<br>4) FAP43 is compensated by FAP244 in *C. reinhardtii*, so FAP44 is lost only in *fap43 fap244* doublet mutants[24]<br>5) Cryo-ET and sub-tomogram averaging of *fap44* and *fap43 fap244* double mutant axonemes shows loss of T/TH complex near IDA*f* motor domains[24,25]<br>6) Our cryo-EM maps of T/TH show a stellate density attached to the IDA*f* motors that closely resembles an AlphaFold2 model of a FAP43/FAP44 complex<br>7) Placement of N-terminal FAP43 and FAP44 β-propeller pairs predicted by the stellate structure are supported by AlphaFold2 models of IDA*f* and T/TH subcomplexes
	MOT7	Cre01.g038750	1) MOT7 is missing or reduced in *C. reinhardtii* and *Leishmania fap44* mutant axonemes [24,103]<br>2) FAP44 co-immunoprecipitates with MOT7[12]<br>3) Sub-tomogram averages show MOT7 labeled with strep-Au localizes to T/TH[12]<br>4) MOT7 ortholog co-purifies with IDA*f* in *Ciona intestinalis*[12]<br>5) FAP43/44 alone do not explain a small density at the vertex of the T/TH stellate structure<br>6) AlphaFold2 model of FAP43/FAP44/MOT7 positions MOT7 inside this extra density observed in our cryo-EM maps
MIA complex	MIA1 (FAP100)<br><br>MIA2 (FAP73)	Cre16.g689600.t1.2<br><br>Cre02.g103950.t1.2	1) MIA1 is required for MIA2 association with the axoneme[21]<br>2) MIA1 and MIA2 crosslink with IDA*f* subunits[21]<br>3) IDA*f* docking is greatly reduced in *mia oda* doublet mutants[21,33]<br>4) Cryo-ET of MIA mutants show missing density near the distal end of IDA*f*[21]<br>5) AlphaFold2 predicts a branched coiled-coil structure for the MIA1/MIA2 complex, consistent with density connecting IDA*f* to ODA-2 and FAP189<br>6) Interactions between MIA and IDA*f* are supported by subcomplex modeling with AlphaFold2<br>7) MIA1 crosslinks with FAP57 and FAP189[21], consistent with our localization
FAP57 homo-dimer	FAP57	Cre04.g217914.t1.1	1) FAP57 orthologs are only found in organisms that assemble IDAs[23]<br>2) Loss of IDA*d* and IDA*g* in *fap57*/*bop2* mutants[23,104,105]<br>3) FAP57 co-purifies with IDA*g*[23]<br>4) FAP57 homodimer identified by sidechain density of a coiled coil underneath IDA*g* and IDA*d*<br>5) FAP57 coiled coil localizes near the C-terminus of FAP43/44 and CCDC96/113, consistent with proximity labelling studies in *Tetrahymena*[26,84]<br>6) *fap57*/*bop2* mutants also lack a structure near the distal end of IDA*f*[23,104]<br>7) Strep-Au labeling localizes the N-terminus of FAP57 near the distal end of IDA*f*[23]<br>9) FAP57 coimmunoprecipitates with MIA1[21]

			10) AlphaFold2 predicts the N-terminus of a FAP57 homodimer to form two pairs of β-propellers followed by a long coiled-coil. This domain architecture fits the density near the distal end of IDA*f* and MIA in our cryo-EM maps
FAP189 homo-dimer	FAP189	Cre13.g584400.t1.2	1) Mutation of *CFAP58* causes defects in mammalian flagella formation[106]<br>2) *C. reinhardtii* contains 3 paralogs of *CFAP58* called *fap58*, *fap189*, and *mbo2* that encode long coiled coil proteins consistent with the length of an L-shaped molecular ruler on the doublet microtubule surface<br>3) Cryo-EM maps of the L-shaped coiled coil near IDA*b* are best fit by a C-terminal fragment of a FAP189 homodimer<br>4) FAP189 cross-links with MIA1, consistent with our placement of both proteins[21]
CCDC96/113	CCDC96 (FAP184)<br><br>CCDC113 (FAP263)	Cre03.g160050.t1.2<br><br>Cre17.g717150.t1.2	1) CCDC96 and CCDC113 are mutually required for localization to the axoneme in *Tetrahymena*[84]<br>2) *CCDC96* and *CCDC113* mutant axonemes lack a structure between the N-DRC, RS3S, and IDA*g* in *Tetrahymena*[84]<br>3) Our cryo-EM maps of this region show a coiled coil crossing perpendicularly over CCDC39/40 and FAP189 before folding back on itself<br>4) AlphaFold2 model of CCDC96/113 complex is a coiled coil with its N-terminal half folded back on itself<br>5) This model places the C-terminal portion of CCDC96/113 underneath RS3S, which is consistent with the proximity labeling of FAP251 by C-, but not N-terminally tagged CCDC96/113[84]
	FAP299	Cre11.g482200.t1.2	1) FAP299 is reduced in *CCDC96* and *CCDC113* mutant axonemes[84]<br>2) Predicted FAP299 structure matches a globular density on top of CCDC96/113<br>3) AlphaFold2 model predicts FAP299 to interact with the region of CCDC96/113 expected from their relative positions in the cryo-EM maps
N-DRC	DRC3	Cre12.g531500.t1.2	1) *drc3* mutants form an N-DRC complex that only lacks DRC3[34]<br>2) Subtomogram averages of *drc3* mutant axonemes show loss of the L1 protrusion that links the N-DRC to IDA*g*[34]<br>3) AlphaFold2 model of DRC3 shows distinctive arrangement of LRR and 3-helical bundle domains that matches our cryo-EM maps of the L1 protrusion<br>4) Arrangement is consistent with the locations of the N- and C-termini of DRC3 identified by strep-Au labels and cryo-ET[107]
	DRC5<br><br>DRC6	Cre01.g045350.t1.2<br><br>Cre12.g515200.t1.2	1) Both DRC5 and DRC6 are specifically lost in *drc5* mutants[30,108,109]<br>2) *drc5* and *drc6* both encode a single LRR domain, but the LRR of DRC5 is larger by ~68%<br>3) Our cryo-EM map of the central linker shows density for a LRR surrounding the DRC1/2/4/4 helical bundle that is consistent with the size of DRC5<br>4) AlphaFold2 model predicts DRC5 to bind the DRC1/2/4/4 helical bundle in the location expected from our cryo-EM maps<br>5) C-terminus of DRC5 in the atomic model is consistent with location of C-terminal strep-Au tag[27,28]<br>6) Subtomogram averages of *drc5* mutant axonemes show loss of density near the distal lobe, in addition to the region occupied by DRC5[30]<br>7) AlphaFold2 model of DRC6 fits our cryo-EM maps of the region near the distal lobe<br>8) The lysine-rich, unstructured N-terminus of DRC6 points toward the neighboring DMT, consistent with the proposed electrostatic interaction between the N-DRC and polyglutamylated tubulin[29]
	DRC7	Cre14.g612700.t1.2	1) Subtomogram averages of *drc7* mutant axonemes show a partial N-DRC complex lacking the OID linker and distal lobe sub-structures[27]<br>2) Mass spectrometry of *drc7* axonemes show only the specific absence of DRC7[27]<br>3) Our cryo-EM maps show density consistent with a β-propeller, papain-like Cys protease, and MORN repeat, consistent with the AlphaFold2-predicted domain organization of DRC7<br>4) The identified location puts DRC7 in direct contact with DRC3 and DRC4, consistent with an N-DRC subcomplex formed by DRC3, DRC4, DRC7 and DRC11[34]<br>5) The OID linker sub-structure of the N-DRC is missing or reduced in wildtype *Tetrahymena* and vertebrate axonemes, suggesting this region is not widely conserved[31,32,84,110]<br>6) The N-terminal LRR and β-propeller of DRC7 are only conserved within Viridiplantae (green plants), and mostly within green algae<br>7) Connected to the β-propeller of DRC7, we observed an extra density on the core of ODA-3 in our cryo-EM maps, which is the correct size and shape for the N-terminal LRR<br>8) AlphaFold2 model of the DRC7 LRR domain bound to IC1/LC2/LC9 of the ODA fit our observed densities
	DRC8<br><br>DRC11	Cre16.g676645.t1.1<br><br>Cre07.g325762.t1.1	1) DRC8 and DRC11 are lost in *drc11* mutants[27]<br>2) DRC11 co-sediments with DRC1/2/4 in *drc5* mutants, suggesting a close association[109]<br>3) Subtomogram averages of *drc11* mutants show loss of density in the proximal lobe[27]<br>4) Our cryo-EM maps of the proximal lobe show the core DRC1/2/4/4 helical bundle bound by additional proteins

			5) DRC8 is a small CaM-like protein, and DRC11 is composed of a C-terminal P-loop NTPase domain with two IQ motifs in its N-terminal half<br>6) AlphaFold2 model predicts association of DRC11 with DRC8/CaM and a helical bundle of DRC2/4/4; DRC1 bends back toward DRC5<br>7) Fit of the model into our cryo-EM map positions lysine-rich regions of DRC2, DRC4, and DRC11 at the interface with the neighboring DMT, consistent with the proposed electrostatic interaction between the N-DRC and polyglutamylated tubulin[29]<br>8) This model is consistent with N-terminal strep-Au tags of DRC1, DRC2 and DRC4[28]<br>9) The resolutions of our cryo-EM maps are too low to distinguish between DRC8 and other CaM-like proteins that co-purify with the N-DRC[28]
	DRC9<br><br>DRC10	Cre01.g025450.t1.2<br><br>Cre02.g141350.t1.2	1) DRC9 and DRC10 are reduced in *pf3/drc1*, *ida6/drc2*, and *pf2/drc4* mutant axonemes, but are retained at wildtype levels in *drc3*, *drc7*, and *drc11*[27,34,41,109]<br>2) Our cryo-EM maps show an unidentified coiled coil extending from the baseplate bulb past the helical bundles of DRC3 – DRC9 and DRC10 are the only unplaced N-DRC subunits that encode coiled coils<br>3) AlphaFold2 model of DRC9/10 subcomplex predicts a long coiled coil with an N-terminal helical bundle consistent with the baseplate bulb<br>4) Position is consistent with additional AlphaFold2 model subcomplexes of DRC1/9/10 and DRC3/9/10 that fit our cryo-EM maps of the N-DRC
Radial spoke 3S (RS3S)	FAP61<br><br>FAP251 (CaM-IP4)		1) FAP61, FAP91, and FAP251 form a stable complex independent of ODA, IDA*f*, central apparatus, and radial spokes 1 and 2[36]<br>2) RS3S is lost by knockdown of FAP61, but not FAP91[35]<br>3) Our cryo-EM maps of RS3S show a pair of β-propellers on the DMT surface that fits the AlphaFold2 model of FAP251<br>4) On top of the β-propellers, the AlphaFold2 model of FAP61 matches a bilobed globular density<br>5) These positions of FAP61 and FAP251 are consistent with the missing densities of *fap61* and *fap251* mutant axonemes in *Tetrahymena* [111]
Single-headed IDAs	actin<br><br>centrin<br><br>p28<br><br>p38<br><br>p44<br><br>IDA*a* (DHC6)<br><br>IDA*b* (DHC5)<br><br>IDA*c* (DHC9)<br><br>IDA*d* (DHC2)<br><br>IDA*e* (DHC8)<br><br>IDA*g* (DHC7)	Cre13.g603700.t1.2<br><br>Cre11.g468450.t1.2<br><br>Cre12.g494800.t1.1<br><br>Cre03.g186300.t1.2<br><br>Cre08.g374650.t1.1<br><br>Cre05.g244250.t1.2<br><br>Cre02.g107050.t1.1<br><br>Cre02.g141606.t1.1<br><br>Cre09.g392282.t1.1<br><br>Cre16.g685450.t1.1<br><br>Cre14.g627576.t1.1	1) Gene for each heavy chain determined by mass spectrometry of purified IDAs[38]<br>2) The locations of the single-headed IDAs within the 96-nm repeat has been previously determined by comparing subtomogram averages of *ida4*, *ida5*, *ida9*, *ida10*, and *pf3/drc1* mutant axonemes[3,39]<br>3) All single-headed IDAs co-purify with actin, but only IDAs *a*, *c,* and *d* associate with p28, and only IDAs *b*, *e* and *g* with centrin[17,112,113]<br>4) Our cryo-EM maps of IDA*a* and IDA*d* show a similar arrangement of an actin monomer and p28 homodimer bound by the N-termini of the heavy chains, similar to that of IDA*c*[14]<br>5) AlphaFold2 models of actin and p28 with the N-termini of IDA*a*, *c* and *d* agree with the cryo-EM maps<br>6) IDA*d* co-purifies with p38 and p44[114,115]<br>7) AlphaFold2 model of p38/p44 complex closely resembles density attached to the base of IDA*d* in our cryo-EM maps<br>8) AlphaFold2 models show a similar arrangement of actin and centrin bound to the N-termini of IDA*b*, *e* and *g*, which agree with our cryo-EM maps of these IDAs

Supplementary Table 3 | External proteins identified in the 96-nm modular repeat of the human doublet microtubule. MIPs are listed in[64]. One additional MIP, RIBC1, was identified in the current work.

Axonemal complex	Axonemal protein	UniProt ID	*C. reinhardtii* ortholog(s)	Mass in kDa / (Residues)
External coiled coils	CCDC39	Q9UFE4	FAP59	109.9 (941)
	CCDC40	Q4G0X9	FAP172	130.1 (1142)
	CCDC96	Q2M329	FAP184	62.7 (555)
	CCDC113	Q9H0I3	FAP263	44.2 (377)
	CFAP299	Q6V702	FAP299	26.9 (233)
	CFAP57	Q96MR6	FAP57	145.0 (1250)
	CFAP58 (and/or CCDC146)	Q5T655	FAP58/FAP189/MBO2	103.4 (872)
ODA	DNAH5	Q8TE73	γ-HC	529.0 (4,624)
	DNAH9	Q9NYC9	β-HC	511.9 (4,486)
	DNAI1	Q9UI46	IC1	79.2 (699)
	DNAI2	Q9GZS0	IC2	68.8 (605)
	DNAL1	Q4LDG9	LC1	21.5 (190)
	DYNLRB1	Q9NP97	LC7a	10.9 (96)
	DYNLRB2	Q8TF09	LC7b	10.9 (96)
	DYNLL1 (and/or DYNLL2)	P63167	LC8	10.4 (89)
	DYNLT2B (and/or DYNLT2, DYNLT4)	Q8WW35	LC2	16.1 (142)
	DYNLT1 (and/or DYNLT3)	P63172	LC9	12.4 (113)
	NME9	Q86XW9	LC3	36.9 (330)
ODA-DC	ODAD1 (CCDC114)	Q96M63	DC2	75.0 (670)
	ODAD2 (ARMC4)	Q5T2S8	DC1	115.7 (1044)
	ODAD3 (CCDC151)	A5D8V7	--	69.1 (595)
	ODAAD4 (TTC25)	Q96NG3	--	76.7 (672)
	ODAD5 (Calaxin, EFCAB1)	Q9HAE3	--	24.5 (211)
IDA*f*	DNAH2	Q9P225	*f*β	507.7 (4,427)
	DNAH10	A0A669KB38	*f*α	528.8 (4,589)
	DNAI3 (WDR63)	Q8IWG1	IC140	102.9 (891)
	DNAI4 (WDR78)	Q5VTH9	IC138	94.6 (848)
	DNAI7 (LAS1)	Q6TDU7	IC97	83.2 (716)
	DYNLRB1	Q9NP97	LC7a	10.9 (96)
	DYNLRB2	Q8TF09	LC7b	10.9 (96)
	DYNLL1 (and/or DYNLL2)	P63167	LC8	10.4 (89)
	DYNLT2B (and/or DYNLT2, DYNLT4)	Q8WW35	LC2	16.1 (142)
	DYNLT1 (and/or DYNLT3)	P63172	LC9	12.5 (113)
MIA	CFAP73	A6NFT4	FAP73	35.9 (308)
	CFAP100	Q494V2	FAP100	71.1 (611)
T/TH	CFAP43	Q8NDM7	FAP43	192.0 (1,655)
	CFAP44	Q96MT7	FAP44	213.9 (1854)

Single headed IDAs	DNAH1 (IDA*d*)	Q9P2D7	DHC2	487.5 (4265)
	DNAH3 (IDAc)	Q8TD57	DHC9	470.8 (4116)
	DNAH6 (IDA*g*)	Q9C0G6	DHC7	476.0 (4158)
	DNAH7 (IDA*b/e*)	Q8WXX0	DHC5/DHC8	461.2 (4,024)
	DNAH12 (IDA*a*)	E9PG32	DHC6	454.3 (3960)
	DNALI1	O14645	p28	29.7 (258)
	CETN2	P41208	centrin	19.7 (172)
	ACTA2	P62736	actin	42.0 (377)
	TTC29	Q8NA56	p44	55.1 (475)
	ZMYND12	Q9H0C1	p38	41.8 (365)
RS1/2	CALM1	P0DP23	CAM1	16.8 (149)
	CYB5D1	Q6P9G0	FAP198	26.7 (228)
	DNAJB13	P59910	RSP16	36.1 (316)
	DYDC2	Q96IM9	RSP2	20.6 (177)
	DYNLL1	P63167	LC8	10.4 (89)
	IQUB	Q8NA54	IQUB	92.6 (791)
	LRRC34	Q8IZ02	RSP15	51.3 (464)
	MORN3	Q6PF18	FAP207	27.6 (240)
	NME5	P56597	RSP23	24.2 (212)
	PPIL6	Q8IXY8	RSP12	35.2 (311)
	ROPN1L	Q96C74	RSP11	26.1 (230)
	RSPH1	Q8WYR4	RSP1	35.1 (309)
	RSPH3	Q86UC2	RSP3	63.7 (560)
	RSPH4A	Q5TD94	RSP4	80.7 (716)
	RSPH9	Q9H1X1	RSP9	31.3 (276)
	RSPH14	Q9UHP6	RSP14	38.6 (348)
	SPA17	Q15506	RSP7	17.4 (151)
RS3	CFAP61	Q8NHU2	FAP61	141.3 (1237)
	CFAP91	Q7Z4T9	FAP91	90.0 (767)
	CFAP251	Q8TBY9	FAP251	130.0 (1,149)
N-DRC	DRC1 (CCDC164)	Q96MC2	DRC1	87.1 (740)
	DRC2 (CCDC65)	Q8IXS2	DRC2	57.3 (484)
	DRC3 (LRRC48)	Q9H069	DRC3	61.0 (523)
	DRC4 (GAS8)	O95995	DRC4	56.4 (478)
	DRC5 (TCTE1)	Q5JU00	DRC5	55.6 (501)
	DRC7 (CCDC135)	Q8IY82	DRC7	103.5 (874)
	DRC8 (EFCAB2)	Q5VUJ9	DRC8	29.7 (269)
	DRC9 (IQCG)	Q9H095	DRC9	51.9 (443)
	DRC10 (IQCD)	Q96DY2	DRC10	52.4 (449)
	DRC11 (IQCA1)	Q86XH1	DRC11	95.3 (822)

Note 1. ACTA2 was modeled in each IDA as it is the most abundant Actin isotype present in our mass spectrometry analysis[64]. ACTG1 and ACTC1 were also observed in the mass spectrometry data and could be present in the axoneme.

Supplementary Table 4 | Rationale for the placement of human axonemal proteins. This table only includes proteins that either have no homolog in the *C. reinhardtii* DMT or where the relationship to an *C. reinhardtii* axonemal protein was not well established.

**Complex**	**Protein**	UniProt ID	**Evidence for location**
ODA	NME9	Q86XW9	1) NME9 shares high sequence and structural similarity with *C. reinhardtii* LC3<br>2) AlphaFold2 predicts an interaction between NME9 and β-ODA (DNAH9, DNAH11 and DNAH17) but not with γ-ODA (DNAH5 and DNAH8)<br>3) NME8, a closely related protein, is not present in our sample by mass spectrometry analysis
IDAs	DNAH1 (IDA*d*)	Q9P2D7	1) Contains a unique N-terminal tail (residues P90-T158) that forms a complex with CFAP61 as predicted by AlphaFold2<br>2) Contains a globular domain in the N-terminus (Q163-C397) that fits with the density map<br>3) Phylogenetic analysis indicates that DNAH1 is orthologous to *C. reinhardtii* DHC2 (IDA*d*)[40]
	DNAH3 (IDA*c*)	Q8TD57	1) The AlphaFold2 prediction of the N-terminal tail (residues G54-V129) fits with the density map
	DNAH6 (IDA*g*)	Q9C0G6	1) The only centrin2-binding dynein heavy chain in human<br>2) Phylogenetic analysis indicates that DNAH6 is orthologous to *C. reinhardtii* DHC7 (IDA*g*)[40]
	DNAH7 (IDA*b/e*)	Q8WXX0	1) Phylogenetic analysis shows DNAH3/7/12 are candidates of IDAb/e<br>2) AlphaFold2 prediction of DNAH7 N-terminal tail contains a helix (H118-M133) that fits the density of IDA*e*<br>3) Some bulky sidechains of DNAH7 (W184, H195, F199, R211) fit the density of IDA*e*<br>4) Tentatively assign IDA*b* as DNAH7 because DNAH7 lacks a globular domain or CCDC39/40 binding tail, which is consistent with the density of IDA*b*
	DNAH12 (IDA*a*)	E9PG32	1) Assigned based on sidechain densities of the N-terminal tail<br>2) The N-terminal tail forms a complex with CCDC39/CCDC40 as predicted by AlphaFold2
RS	LRRC34	Q8IZ02	1) Expressed in ciliated cells<br>2) AlphaFold2 model is a good fit for the density<br>3) LRRC23, a closely related protein, is reported to be a RS protein in mouse sperm[116]. Although LRRC23 is present in our mass spectrometry analysis[64], its AlphaFold2 prediction does not agree with the density map.
	MORN3	Q6PF18	1) Expressed in ciliated cells<br>2) AlphaFold2 prediction is a good fit for the density
	SPA17	Q15506	1) Expressed in ciliated cells<br>2) Identified as a homolog of RSP7 by a BLASTP search<br>3) Like RSP7, contains a Dpy-30 motif<br>4) Forms a complex with RSPH3 based on AlphaFold2 predictions<br>5) ROPN1B, a closely related protein, is not present in our sample by mass spectrometry analysis

Note 1. DNAH9 and DNAH11 locate at the distal and proximal regions of the human respiratory axoneme, respectively[117]. We built model for DNAH9 because it's slightly more abundant than DNAH11 in our mass spectrometry analysis.

Note 2. The molecular identities for the light chains of IDA*f* and ODA are not certain. We tentatively assigned DYNLL1, DYNLT2B and DYNLT1 based on their presence in our mass spectrometry analysis and AlphaFold2 predictions. Other paralogs are also possible.

Note 3. DRC8 is an EF-hand containing protein. We assigned four copies of DRC8 at the IQ motifs of DRC9/10/11 based on AlphaFold2 predictions. We cannot rule out that some DRC8 molecules are Calmodulin instead.

Supplementary Table 5 | Structure-verified human axonemal proteins associated with disease. Please note that the list of references is not intended to be exhaustive. Where possible, the first examples linking the gene to disease are included. Abbreviations used in the table: MMAF, multiple morphological abnormalities of sperm flagella; PCD, primary ciliary dyskinesia.

Axonemal complex	Axonemal protein	OMIM	Human disease association
DMT	TUBA1A	602529	Lissencephaly[118]
	TUBB4B	602660	PCD[119]; Leber congenital amaurosis[120]
MIPs	CFAP20	617906	Retinitis pigmentosa[121]
	CFAP21	-	No disease association
	CFAP45	605152	Laterality abnormalities and asthenospermia; mild respiratory symptoms[122]
	CFAP52	609804	Laterality abnormalities[122,123]; mild respiratory symptoms[122]
	CFAP53	614759	Laterality abnormalities[124–126]
	CFAP68	-	No disease association
	CFAP77	-	No disease association
	CFAP90	-	No disease association
	CFAP95	-	No disease association
	CFAP107	-	No disease association
	CFAP126	616119	No disease association
	CFAP141	-	No disease association
	CFAP161	-	No disease association
	CFAP210	-	No disease association
	CFAP276	618682	Charcot-Marie-Tooth disease[127]
	EFHC1	608815	Debated association with juvenile myoclonic epilepsy[128]
	EFHC2	300817	Debated association with juvenile myoclonic epilepsy[129]
	ENKUR	611025	Laterality defects[130]
	FAM166B	-	Meckel-Gruber syndrome[131]
	FAM166C	-	No disease association
	MNS1	610766	Laterality defects and male infertility[132–134]
	NME7	613465	Laterality abnormalities[135]
	PACRG	608427	Leprosy susceptibility[136]
	Pierce 1	614502	No disease association
	Pierce 2	619669	No disease association
	RIBC1	-	No disease association
	RIBC2	-	No disease association
	SPACA9	618552	No disease association
	SPAG8	605731	No disease association
	Tektin 1	609002	Potentially associated with PCD[137]
	Tektin 2	608953	No disease association
	Tektin 3	612683	No disease association
	Tektin 4	-	No disease association
External coiled coils	CCDC39	613798	PCD[138,139]
	CCDC40	613799	PCD[138,140]

	CCDC96	619347	No disease association
	CCDC113	616070	No disease association
	CFAP299	-	No disease association
	CFAP58	619129	MMAF[106,141,142]
**RS1/2**	CALM1/2/3*		No disease association
	CYB5D1	-	No disease association
	DNAJB13	610263	PCD[143]
	DYDC2	-	No disease association
	DYNLL1*	601562	No disease association
	IQUB	-	No disease association
	LRRC34	619037	Joubert syndrome[131]
	MORN3	-	No disease association
	NME5	603575	PCD[144,145]
	PPIL6	-	No disease association
	ROPN1L	611756	No disease association
	RSPH1	609314	PCD[146,147]
	RSPH3	615876	PCD[148]
	RSPH4A	612647	PCD[149–151]
	RSPH9	612648	PCD[149]
	RSPH14	605663	No disease association
	SPA17 (tentative)	608621	No disease association
**RS3**	CFAP61	-	MMAF[152–154]
	CFAP91	609910	MMAF[155]
	CFAP251	618146	MMAF[156–158]
**N-DRC**	DRC1 (CCDC164)	615288	PCD[159,160]; MMAF[161,162]
	DRC2 (CCDC65)	611088	PCD[163,164]
	DRC3 (LRRC48)	618758	No disease association
	DRC4 (GAS8)	605178	PCD[165–167]
	DRC5 (TCTE1)	186975	MMAF[168]
	DRC7 (CCDC135)	618769	No disease association
	DRC8 (EFCAB2)	619617	No disease association
	DRC9 (IQCG)	612477	No disease association
	DRC10 (IQCD)	-	No disease association
	DRC11 (IQCA1)	-	No disease association
**Single-headed IDAs**	DNAH1 (IDA*d*)	603332	PCD[169]; MMAF[170]
	DNAH3 (IDA*c*)	603334	No disease association
	DNAH6 (IDA*g*)	603336	PCD-like[171]; MMAF[172,173]
	DNAH7 (IDA*b/e*)	610061	PCD[174]; MMAF[175,176]
	DNAH12 (IDA*a*)	603340	MMAF[142,177]
	DNALI1	602135	No disease association
	CETN2*	300006	No disease association
	ACTA2*	102620	No disease association
	TTC29	618735	MMAF[178,179]
	ZMYND12	-	Asthma[180]

**IDA*f***	DNAH2	603333	MMAF[181–183]
	DNAH10	605884	MMAF[177,184,185]
	DNAI3 (WDR63),	617968	MMAF[186]
	DNAI4 (WDR78)	619156	No disease association
	DNAI7 (CASC1, LAS1, CFAP94)	616906	Lung tumorigenesis[187]
	DYNLRB1*	607167	No disease association
	DYNLRB2*	607168	No disease association
	DYNLL1 (and/or DYNLL2)*	601562	No disease association
	DYNLT2B*	617353	Skeletal ciliopathy[188]
	DYNLT1 (and/or DYNLT3)*	601554	No disease association
**MIA**	CFAP73	-	No disease association
	CFAP100	-	COPD and lung cancer[189]
**IDA*f* docking**	CFAP57	614259	PCD[105]; Van der Woude syndrome[190]
**T/TH**	CFAP43	617558	MMAF[191]; Hydrocephalus[192]
	CFAP44	617559	MMAF[191,193]
**ODA**	DNAH9 / DNAH11	603330 / 603339	PCD[117,194]
	DNAH5	603335	PCD[195,196]
	DNAI1	604366	PCD[197]
	DNAI2	605483	PCD[198]
	DNAL1	610062	PCD[199]
	DYNLRB1*	607167	No disease association
	DYNLRB2*	607168	No disease association
	DYNLL1 (and/or DYNLL2)*	601562	No disease association
	DYNLT2B*	617353	Skeletal ciliopathy[188]
	DYNLT1 (and/or DYNLT3)*	601554	No disease association
	NME9	618584	No disease association
**ODA-DC**	ODAD1 (CCDC114)	615038	PCD[42,200]
	ODAD2 (ARCM4)	615408	PCD[201,202]
	ODAD3 (CCDC151)	615956	PCD[203,204]
	ODAD4 (TTC25)	617095	PCD[205]
	ODAD5 (Calaxin, EFCAB1)	619564	PCD[206]

*protein(s) also found in non-axonemal complexes.

Section 2. Figures related to cryo-EM processing of the *C. reinhardtii* axoneme.

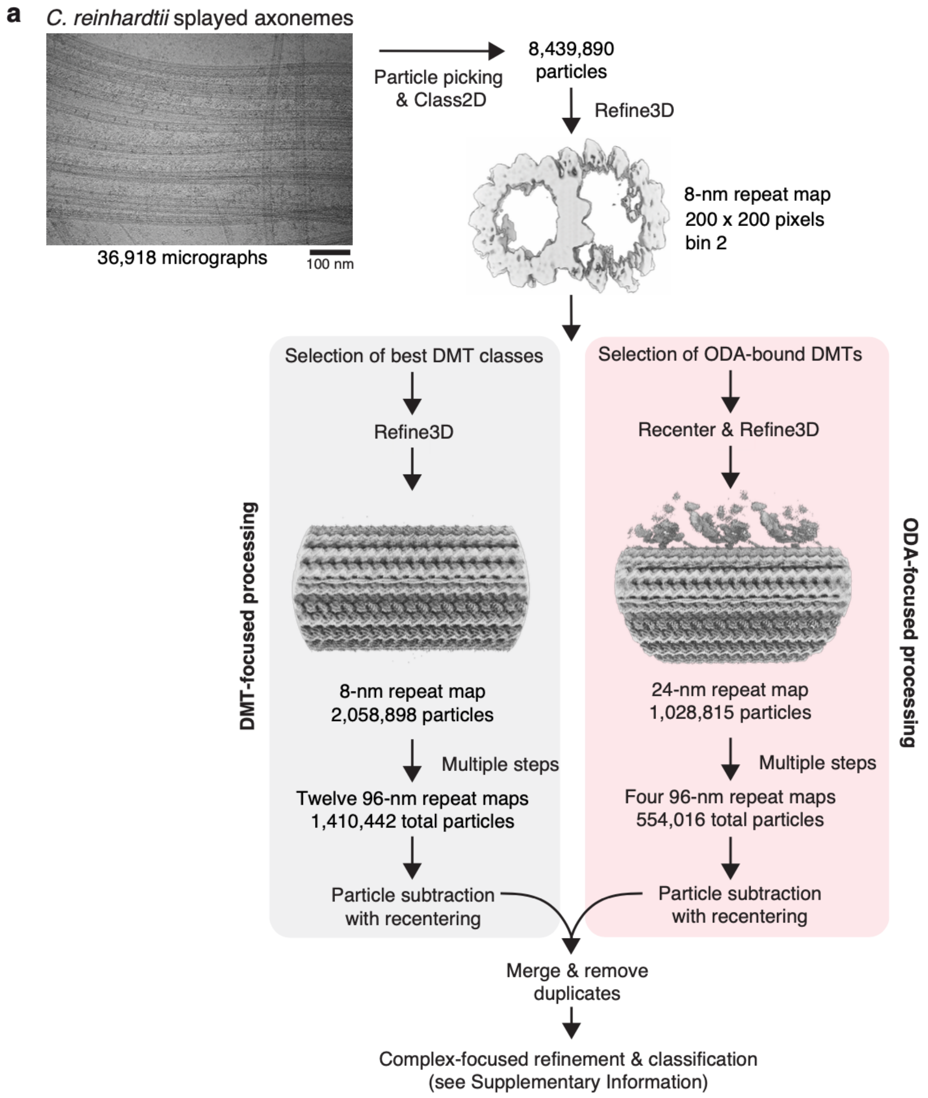

b

Complex	Particle number	Resolution (Å)	Modeling approach
DMT A-tubule refinements	203,786-253,131	3.2-3.5	6U42, DN, AF2
DMT B-tubule refinements	125,499-162,111	3.2-3.8	6U42, DN, AF2
ODA1 core / IDA*f* core	68,572	7.7	7KZM, AF2
ODA2 core / MIA	87,422	6.4	7KZM, AF2
ODA3 core / DRC7	148,414	4.7	7KZM, AF2
ODA β-HC motor	79,172	7.6	AF2, SW
Distal protrusion	261,585	3.3	DN, AF2
N-DRC baseplate	165,859	3.2	7JU4, DN, AF2
N-DRC linker	44,283-163,123	6.2-8.5	AF2
N-DRC closed - box 600	34,410	7.8	--
N-DRC open - box 600	41,199	7.8	--
Tetherhead / inner junction	229,291	3.0	DN
Tetherhead / IDA*f* motors	27,936	16.7	AF2
IDA*a* base and motor / RS1	20,785	4.5-14.1	7JTK, 7JTS, AF2, SW
IDA*b* base	41,996	6.6	AF2
IDA*c* base and motor / RS2	45,965	7.5	7JTK, 7JU4, AF2, SW
IDA*e* base	44,255	4.1	AF2
IDA*g* base and motor	34,962-67,754	3.7-8.6	AF2, SW
IDA*d* base and motor / RS3S	34,480-67,754	6.2-8.7	AF2, SW
IDA*f* core	99,014-196,797	8.4-10.2	AF2

Supplementary Fig. 1 | Overview of the processing strategy used to generate a composite map and atomic model of the *C. reinhardtii* doublet microtubule (DMT). **a**, DMT particles – each covering 8 nm – were selected from 36,918 micrographs and subjected to two-dimensional classification. A subset of 8,439,890 particles were refined to generate a consensus structure that formed the basis of all downstream processing steps. Two separate processing strategies were employed – one focused on the DMT (left, gray box) and the other on outer dynein arm (ODA)-bound particles (right, pink box). From the 8-nm particles, multiple maps corresponding to different registries of the 96-nm repeat were generated. Individual complexes were subsequently computationally extracted from the 96-nm maps from both strategies, merged, and duplicates removed. The resulting particle stacks were then used for focused refinement and classification. The final maps were merged to form a single composite map. Processing schemes for each major axonemal complex are provided in Supplementary Fig. 1-8. **b**, Table summarizing the number of particles used to reconstruct each *C. reinhardtii* axonemal complex, the resolution achieved, and the modeling approach used to interpret the maps. Abbreviations: DN, *de novo* model building; AF2, AlphaFold2-enabled model building; SW, homology modeling using SWISS-MODEL. If a pre-existing PDB entry was used, its 4-character accession code is provided. Further details on the modeling of individual proteins are provided in Supplementary Table 2.

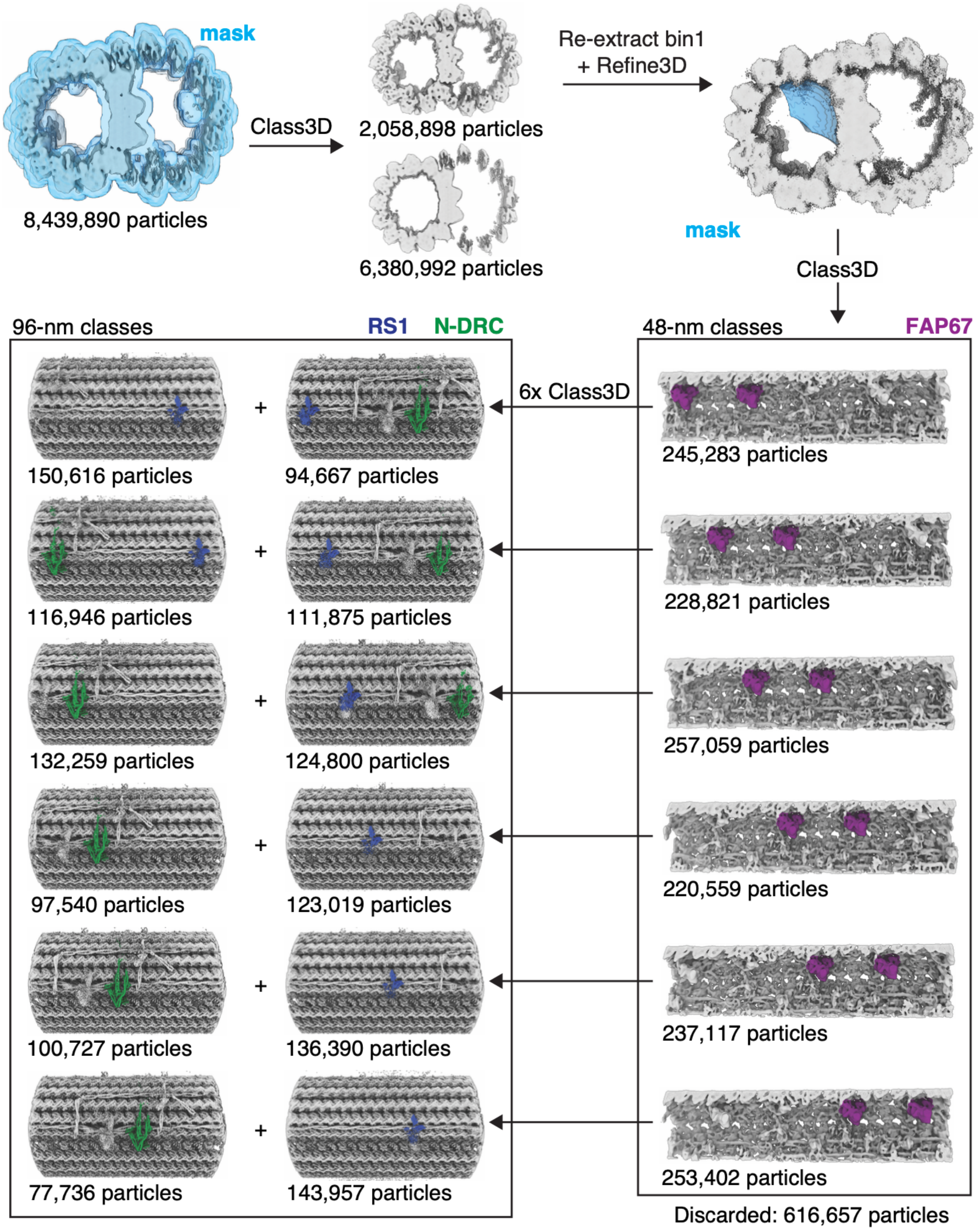

Supplementary Fig. 2 | Processing strategy for the 96-nm modular repeat. 8,439,890 "8-nm particles" were subjected to 3D classification to isolate particles with well-defined doublet microtubule density. 2,058,898 retained particles were classified based on microtubule inner protein (MIP) densities near the A-tubule seam that have 48-nm periodicity. This classification resulted in six different classes that were distinguished based on the 8-nm shifts of FAP67. 616,657 particles were discarded. Each class was subsequently separated into two classes corresponding to the different halves of the 96-nm external repeat.

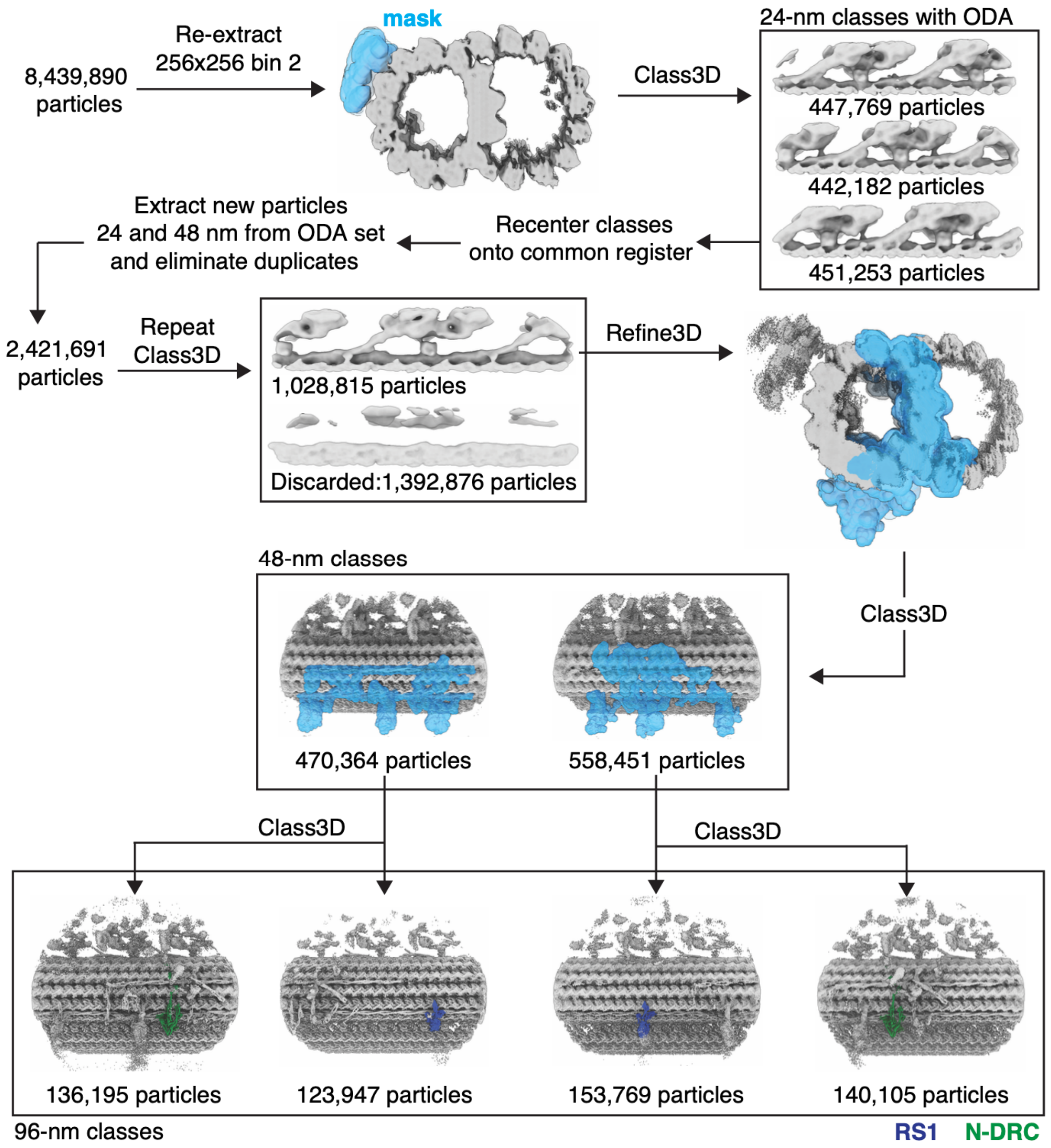

Supplementary Fig. 3 | Processing strategy for outer dynein arms (ODA). 8,439,890 "8-nm particles" were subjected to 3D classification using a mask applied above protofilaments A07–A08 of the doublet microtubule (DMT). This produced 3 classes containing ODA-bound particles, corresponding to the 3 possible registers of the ODA. These classes were recentered onto a common register, and duplicated particles were eliminated. In order to maximize the amount of data retained at this step, the coordinates of the ODA-containing particles were used to extract new particles 24 and 48 nm along the DMT axis, since ODAs dock onto the DMT every 24 nm. The resulting 2,421,691 particles were again subject to 3D classification, yielding one class with strong ODA density, and a discarded class with weak ODA density. Two additional rounds of 3D classification were then used to generate 48- and 96-nm classes.

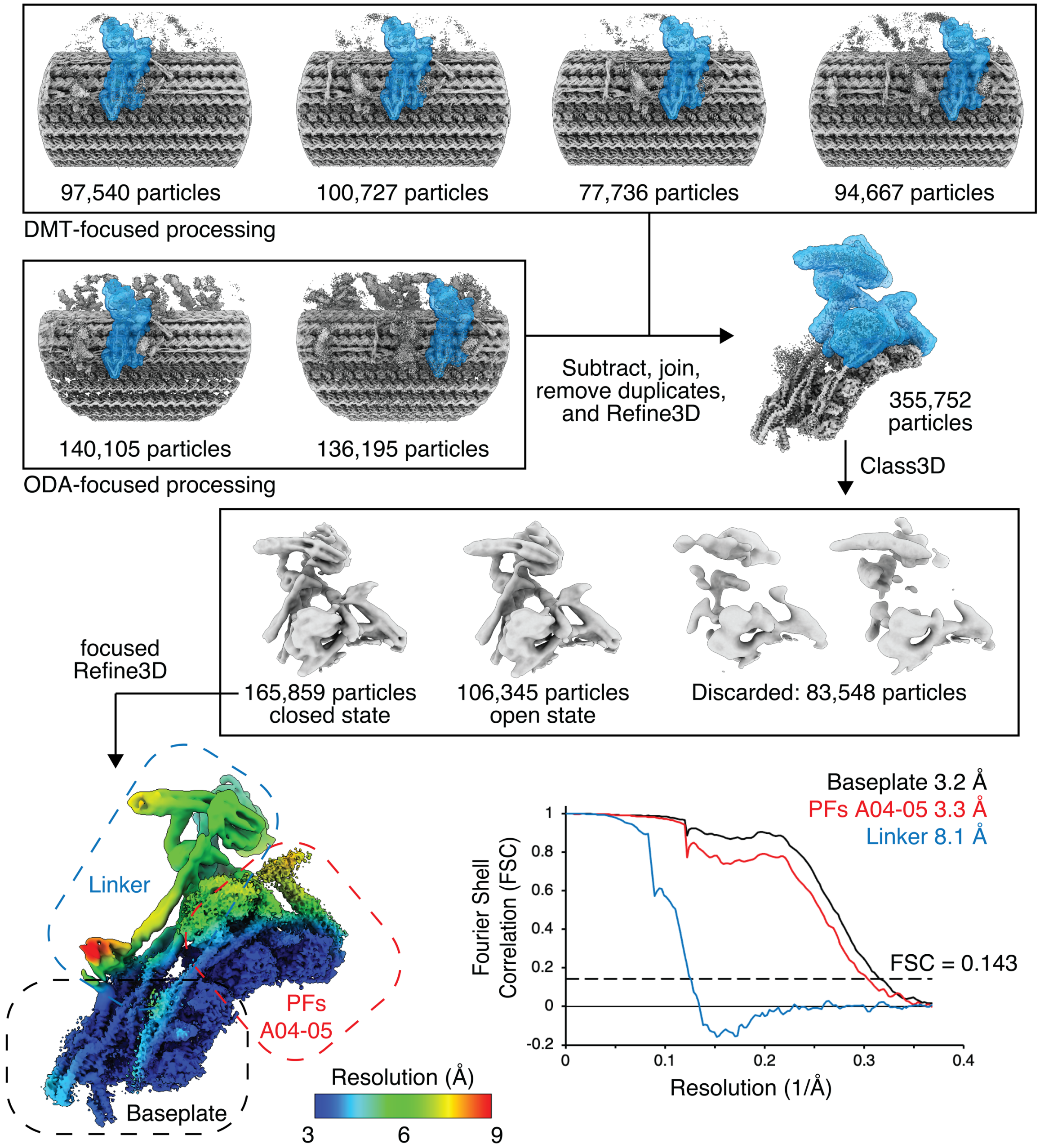

Supplementary Fig. 4 | Processing strategy for the nexin-dynein regulatory complex (N-DRC). Particle subtraction with recentering was completed for 4 registries of the doublet-centered 96-nm repeat and 2 registries of the ODA-centered 96-nm repeat using a mask (blue) covering the baseplate and the lower half of the linker. These particles were combined, and duplicated particles were eliminated. After 3D refinement, the particles underwent 3D classification using a mask over the lower half of the linker and nearby proteins excluding tubulin. Two classes were identified representing open and closed conformations of the N-DRC linker. The more abundant and well-resolved closed state was chosen for focused refinement over the baseplate, linker, and protofilaments A04–A05. Highest resolution was obtained for the tubulin and baseplate region of the N-DRC. Refinement of the linker without tubulin yielded an 8.1-Å resolution map.

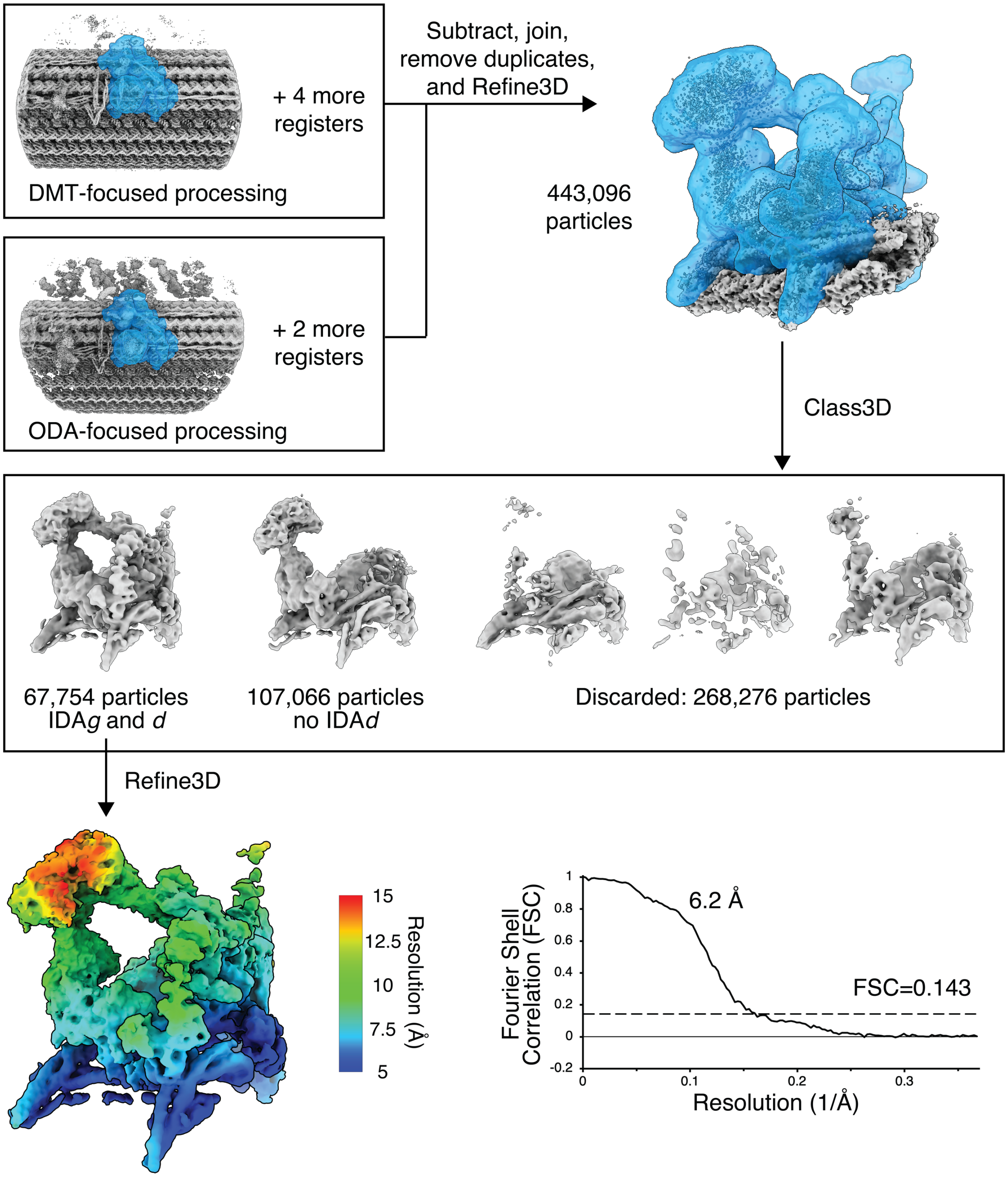

Supplementary Fig. 5 | Processing strategy for radial spoke 3 (RS3S) and the bases of IDA*g*/*d*. Signal subtraction and recentering from the 96-nm repeat maps yielded 443,096 particles after eliminating duplicates. After 3D refinement, density for RS3S and IDA*g*/*d* remained weak, likely due to partial loss of the IDAs and flexibility of RS3S. 3D classification using a mask that excluded tubulin yielded 2 well-resolved classes. The first class contained RS3S, IDA*g*, IDA*d*, and additional density we later identified as p38/44. The second class contained RS3S in an alternative conformation but lacked IDAs and other associated proteins. The first class was chosen for additional 3D refinement to yield a 6.2-Å resolution map.

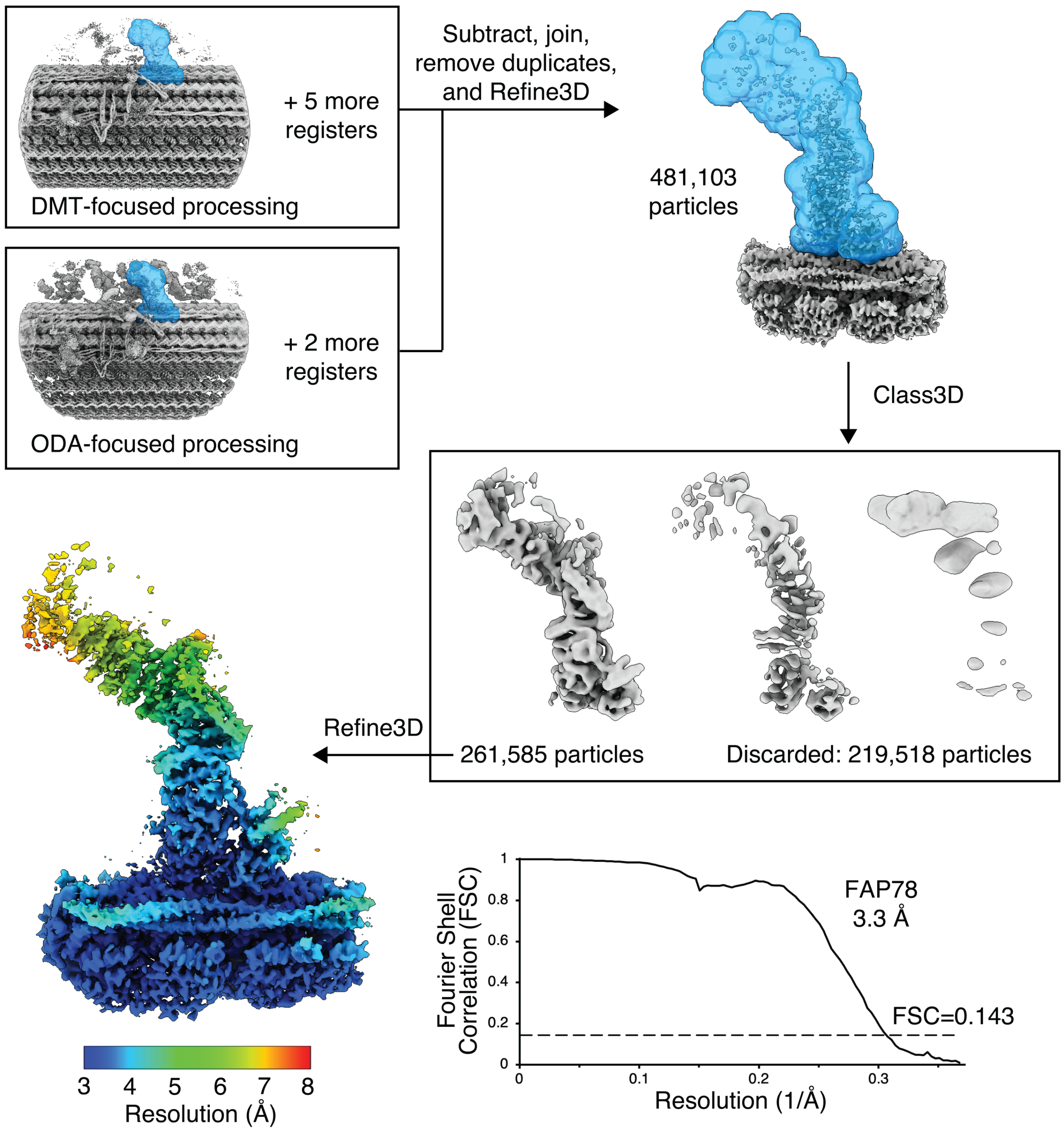

Supplementary Fig. 6 | Processing strategy for the distal protrusion (DP). Signal subtraction and reboxing across the 96-nm repeat maps yielded 481,103 particles after eliminating duplicates. After 3D refinement of all subtracted particles, 3D classification using a mask over the DP, but excluding tubulin, yielded a single class with strong density that contained 261,585 particles. 3D refinement of this class yielded a 3.3-Å resolution map, although the local resolution worsens with distance from the tubulin surface.

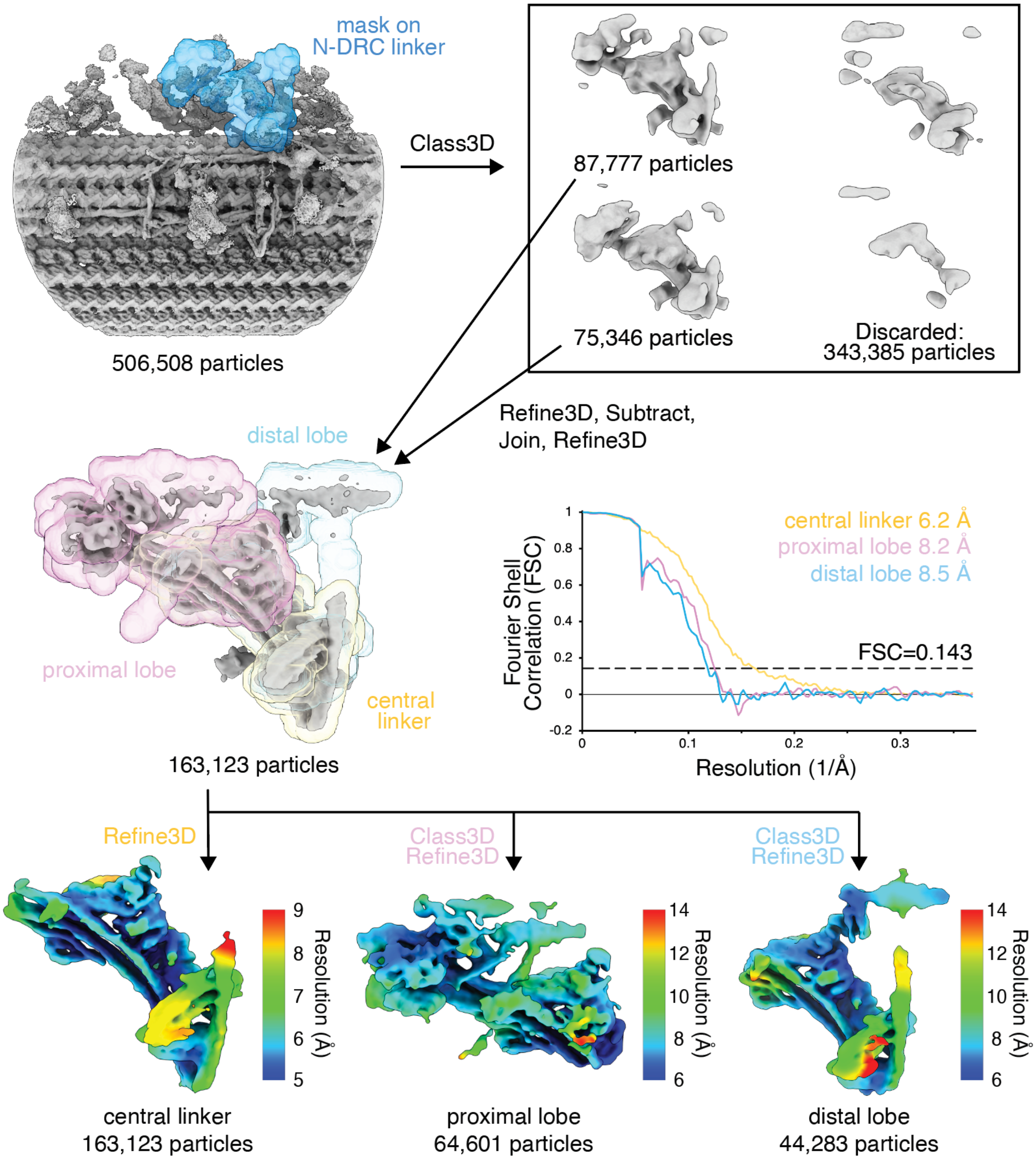

Supplementary Fig. 7 | Processing strategy for the N-DRC linker. 3D classification of the 96-nm maps using a mask over the N-DRC linker yielded two classes with strong linker density. These were separately refined and then subtracted before being joined together, yielding 163,123 particles. 3D refinement produced a map with variable quality. The area of strongest density, the central linker, underwent focused 3D refinement with all 163,123 particles to yield a map of 6.2-Å resolution. The proximal lobe of the linker underwent 3D classification followed by 3D refinement to yield a map of 8.2-Å resolution from 64,601 particles. The distal lobe also underwent 3D classification followed by 3D refinement to yield a map of 8.5-Å resolution from 44,283 particles.

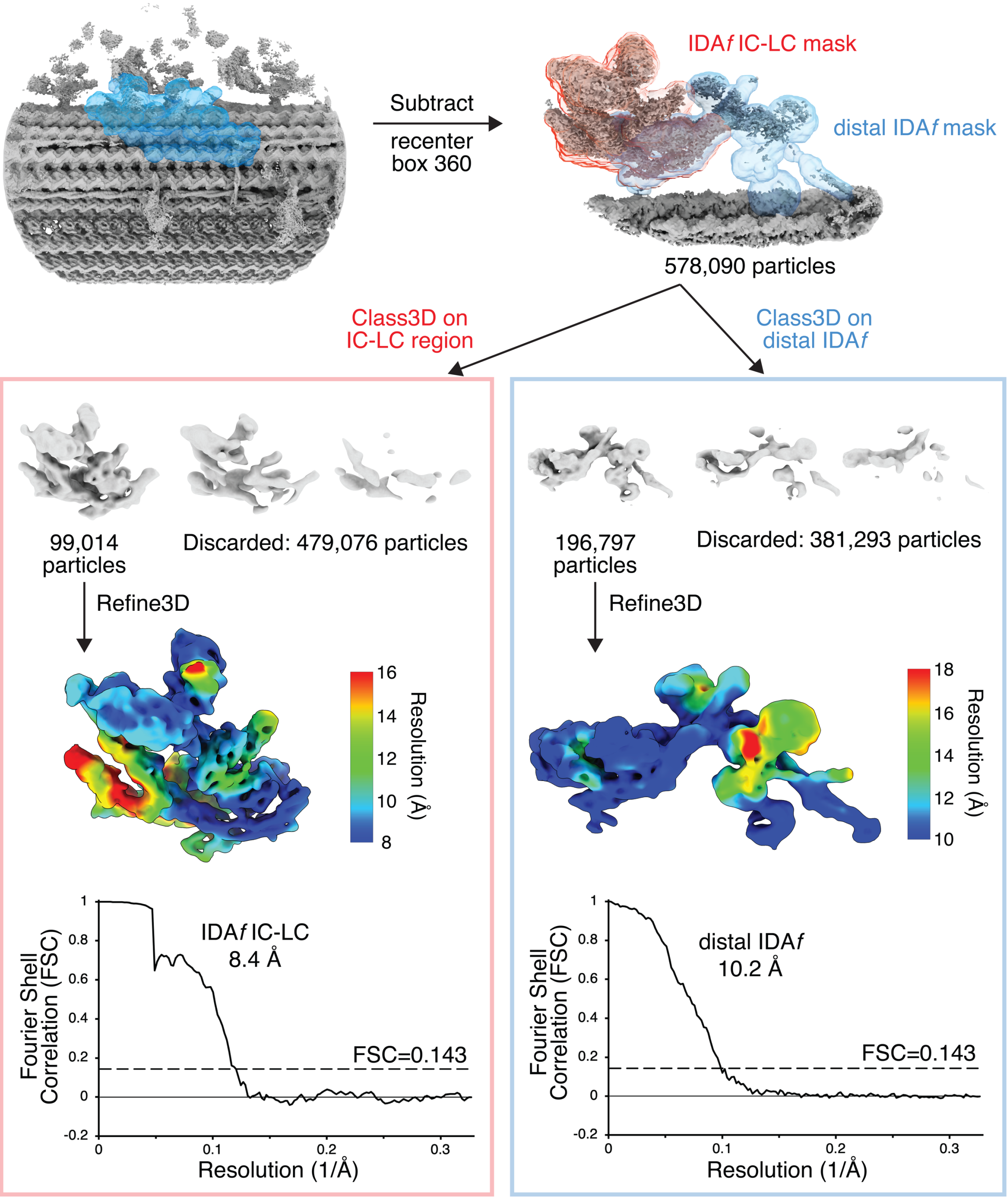

Supplementary Fig. 8 | Processing strategy for the inner dynein arm *f* (IDA*f*) core. Signal subtraction of the 96-nm maps yielded 578,090 particles that, after 3D refinement, yielded a map with nebulous density for IDA*f*. These particles underwent two separate 3D classification jobs that targeted either the proximal, IC-LC region of IDA*f*, or the distal half containing MIA and FAP57, using different masks. For the IC-LC region, 3D classification yielded a single class with strong IDA*f* density containing 99,014 particles. 3D refinement of this particle set yielded a map of 8.4-Å resolution. For the distal region, 3D classification yielded a single class with 196,797 particles displaying density for the four β-propellers of a FAP57 dimer. This set of particles underwent 3D refinement to yield a map of 10.2-Å resolution.

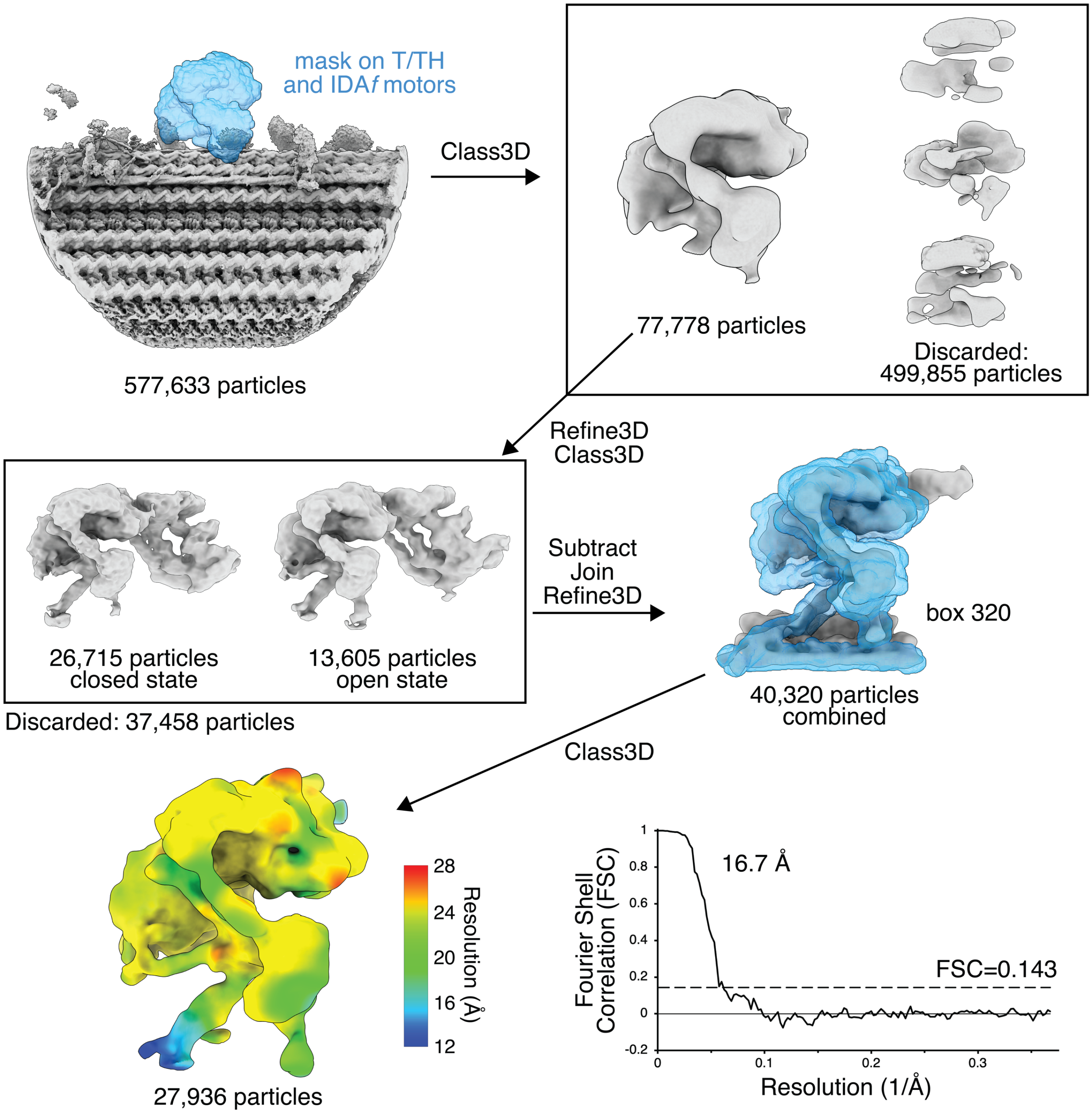

Supplementary Fig. 9 | Processing strategy for the tether/tetherhead (T/TH) complex and associated IDA*f* motors. 3D classification of the 96-nm maps yielded a single class containing 77,778 particles with density for the T/TH and IDA*f* motors. The large number of discarded particles is likely due to loss of IDA*f* and the flexibility of the T/TH complex. After 3D refinement, a second round of 3D classification on the IDA*f* core was used to obtain open and closed states, with 37,458 particles discarded. The remaining 40,320 particles underwent particle subtraction, and the open and closed states of IDA*f* were combined for 3D refinement before a third round of 3D classification to yield 27,936 particles. Focused 3D refinement on T/TH and IDA*f* motors yielded a 16.7-Å resolution map.

Section 3. Figures related to model building of the *C. reinhardtii* axoneme.

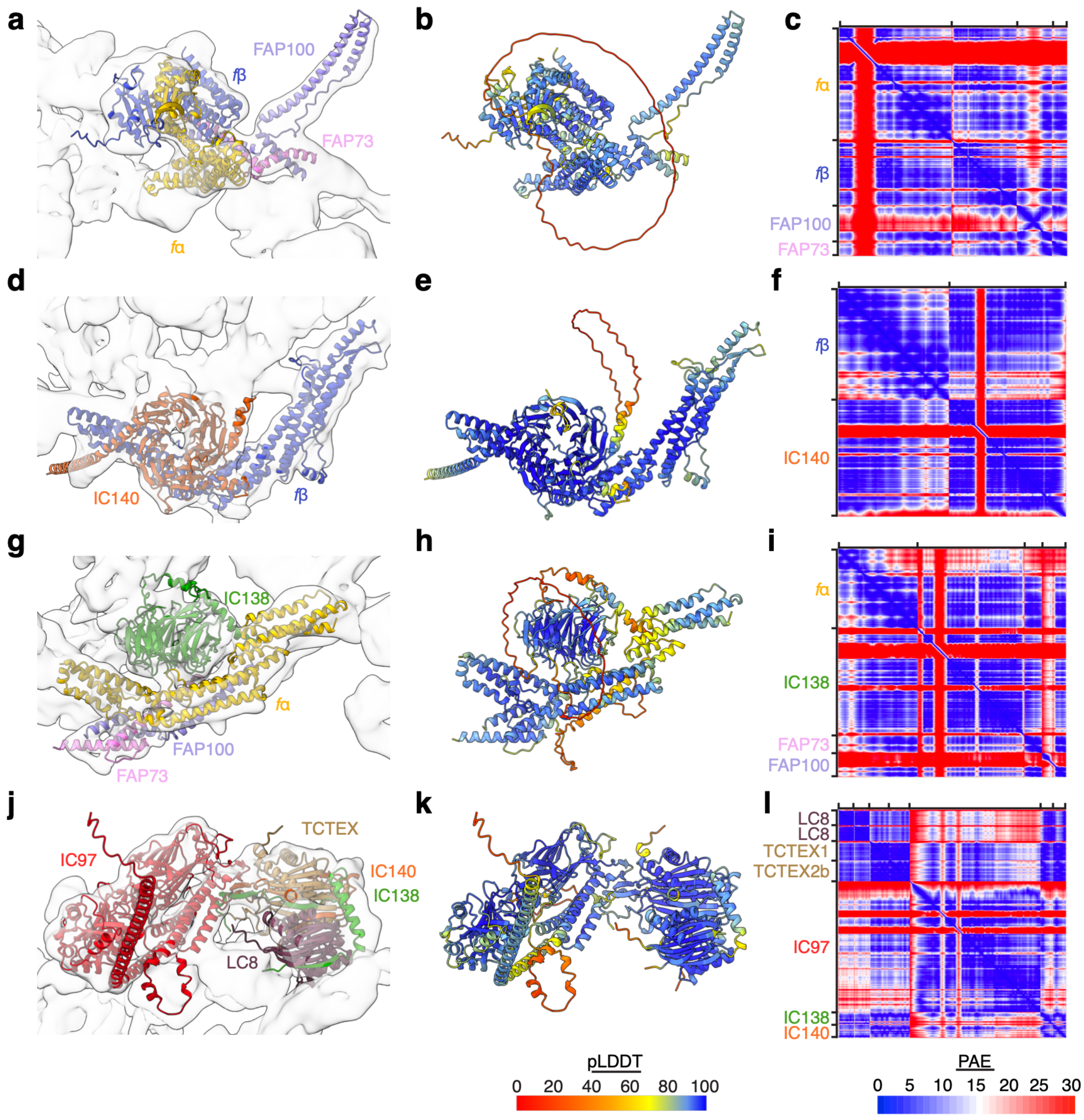

Supplementary Fig. 10 | AI-enabled modeling of IDA*f*. **a**, AlphaFold2 model of the *C. reinhardtii* MIA complex (FAP73/100) fragment with the N-termini of *f*α and *f*β, including the N-terminal dimerization domain, colored by subunit and fit inside our cryo-EM map of IDA*f*. **b**, AlphaFold2 model colored by per-residue confidence score (pLDDT). **c**, Predicted aligned error (PAE) plot showing low alignment error (blue) between portions of all four chains. **d**, AlphaFold2 model of IC140 β-propeller and helical bundles of *f*β heavy chain colored by subunit, and (**e**) colored by pLDDT. **f**, PAE plot showing large regions of low alignment error between IC140 and *f*β, indicating high confidence in the predicted protein-protein interaction. **g**, AlphaFold2 model of the IC138 β-propeller, a fragment of the MIA complex, and *f*α helical bundles colored by subunit, and **h**, colored by pLDDT. IC138 contains unstructured loops. **i**, PAE plot showing regions of low alignment error between all chains. **j**, AlphaFold2 model of LC8, TCTEX, and IC97

with the N-terminal tails of IC138 and IC140 colored by subunit, and (**k**) colored by pLDDT. **l**, PAE plot showing regions of low alignment error for all chains. For all predicted subcomplexes, unstructured loops with low pLDDT were removed from the models fit into the cryo-EM maps.

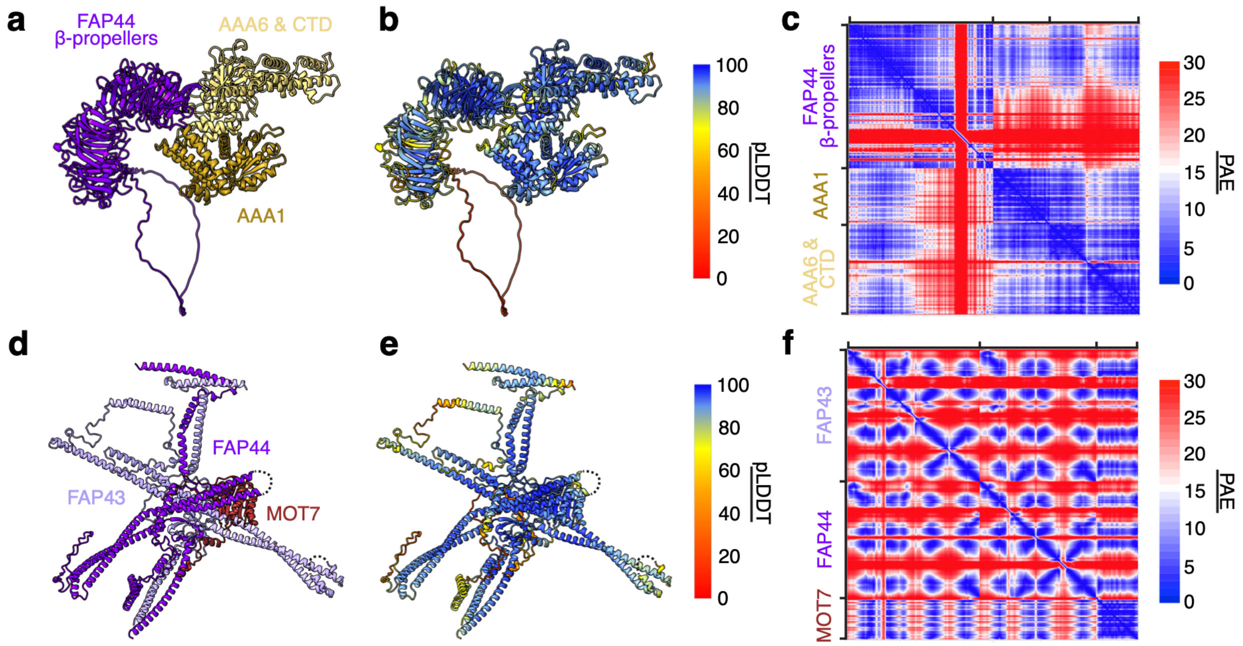

Supplementary Fig. 11 | AI-enabled modeling of the *IDAf* tether/tetherhead (T/TH) complex. **a**, Top-ranked AlphaFold2 model colored by subunit, and **b**, by per-residue confidence score (pLDDT). **c**, Predicted aligned error (PAE) plot of the model shows low error (blue) for the N-terminal β-propeller of FAP44 with the AAA1, AAA6 and C-terminal domains of the *f*α motor. **d**, Top-ranked AlphaFold2 model of the stellate structure colored by subunit, and (**e**) by pLDDT. Due to sequence length limitations, large loops of arms 2 and 3 were replaced with shorter poly-glycine sequences (dashed lines). **f**, Intermittent regions of FAP43 and FAP44 show low PAE with each other and MOT7 consistent with the stellate structure.

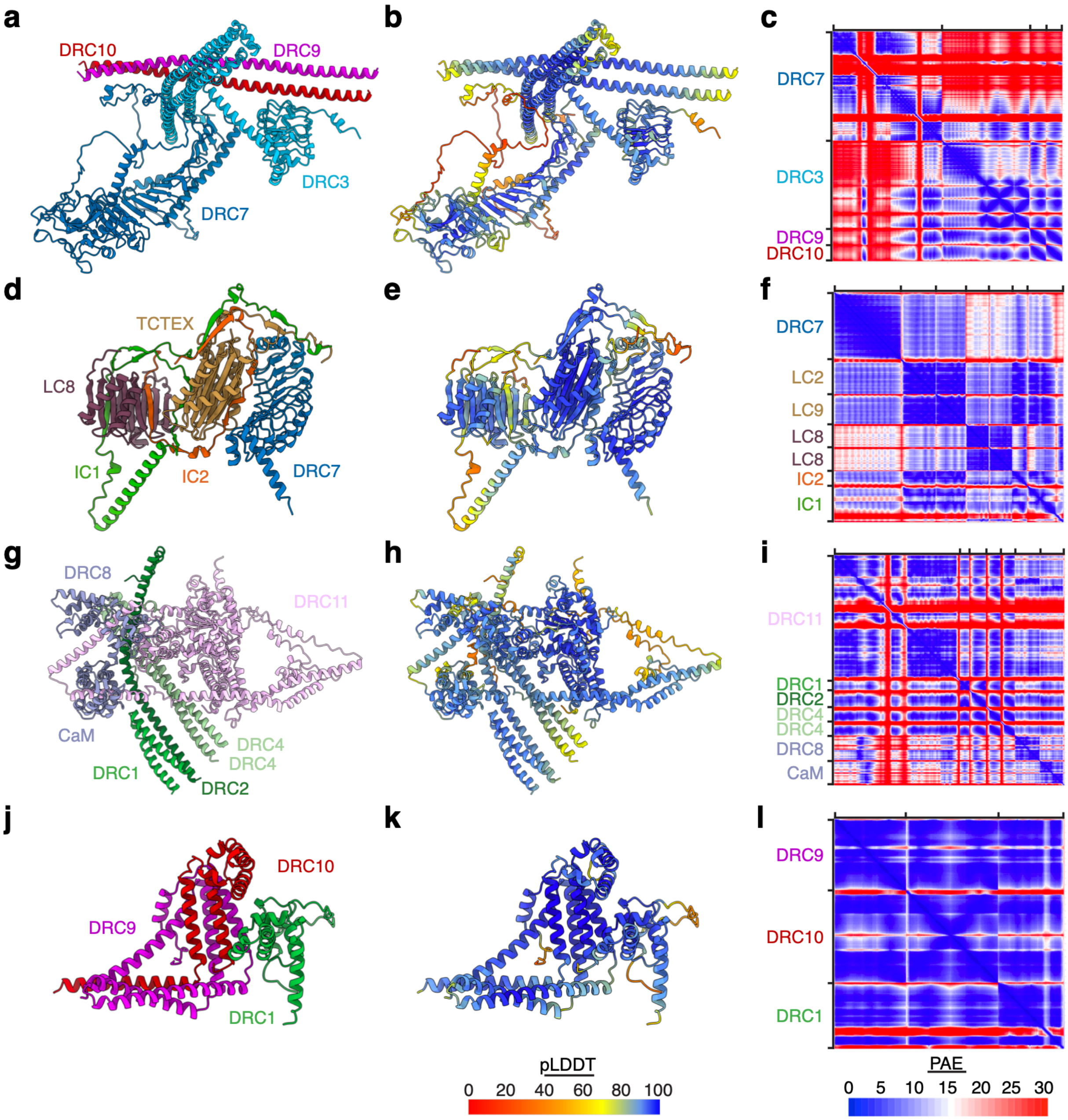

Supplementary Fig. 12 | AI-enabled modeling of the N-DRC. **a**, AlphaFold2 model of DRC3, 7, 9 and 10 colored by subunit, and (**b**) colored by per-residue confidence score (pLDDT). The DRC9/10 coiled coil interacts with both the leucine-rich repeat (LRR) and 3-helix bundle of DRC3. **c**, Predicted alignment error (PAE) plot of AlphaFold2 model showing regions of low alignment error (blue). DRC3, 9 and 10 are well-aligned with each other throughout most of the included sequence, but only a small region of DRC7 is well-aligned with the other 3 chains. **d**, AlphaFold2 model of the connection between the N-terminal LRR of DRC7 and ODA subunits TCTEX, LC8, IC1, and IC2 colored by subunit, and (**e**) colored by pLDDT. **f**, PAE plot showing low alignment error between DRC7, IC1, IC2, and TCTEX (LC2/LC9), supporting our identification of DRC7 as mediating the outer-inner dynein (OID) link. **g**, AlphaFold2 model of DRC11 with the DRC1/2/4/4 helical bundle and DRC8/CaM colored by subunit, and (**h**) colored by pLDDT. **I**, PAE plot showing regions of low alignment error for all 7 chains. **j**, AlphaFold2 model of N-DRC "bulb" composed of part

of DRC1 and the N-termini of DRC9 and 10 colored by subunit, and (**k**) colored by pLDDT. The structure forms a distinctive triangular shape. **l**, PAE plot showing low alignment error for most of this subcomplex.

Section 4. Figures related to cryo-EM processing of the human axoneme.

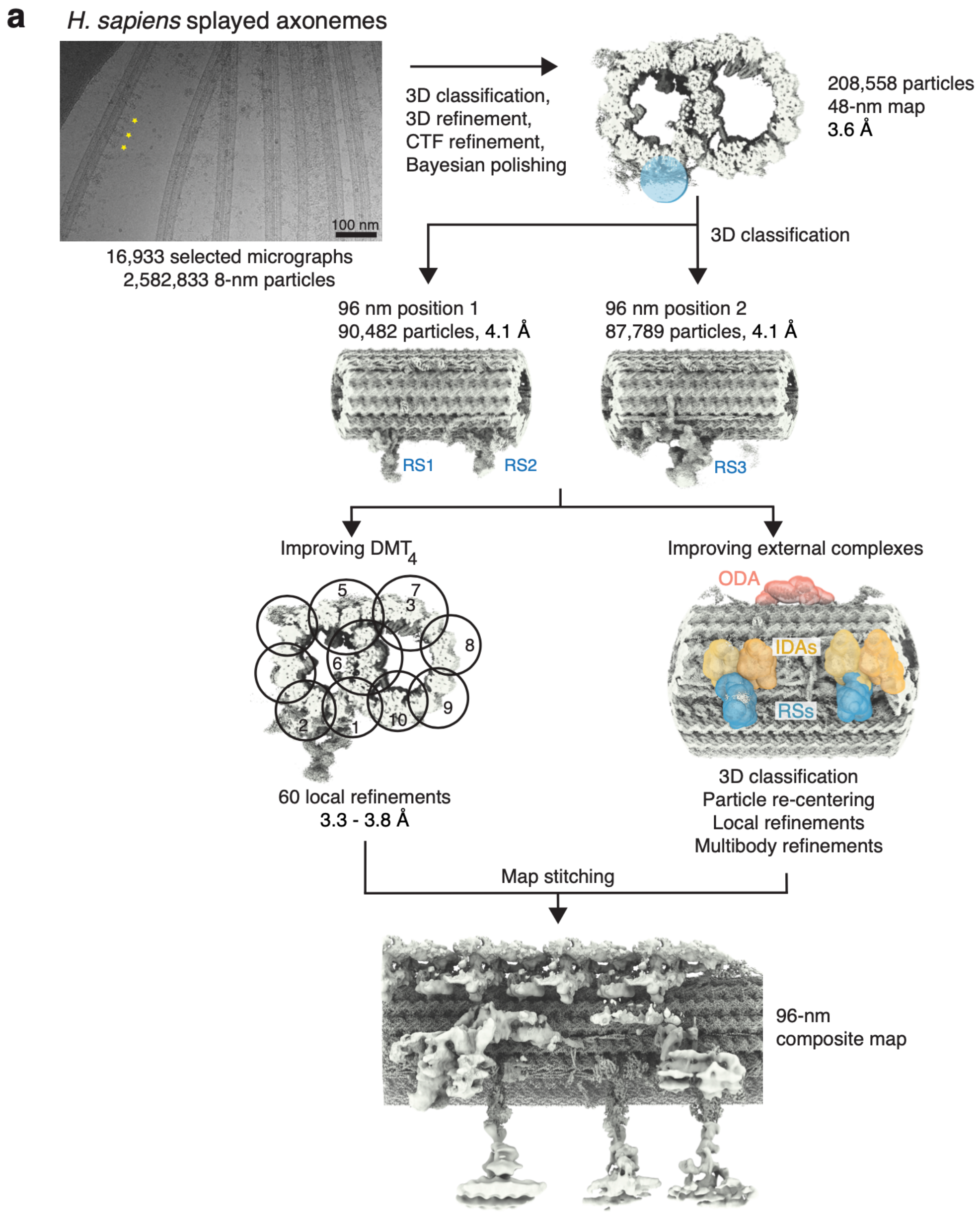

Supplementary Fig. 13 | Processing of the human doublet microtubule (DMT). a, Processing of the human DMT was carried out using a DMT-focused approach with a single registry of the 96-nm repeat. The processing workflow started with 2,582,833 "8-nm particles" selected from 16,933 micrographs. From these particles, a 48-nm map was reconstructed from 208,558 particles with a nominal resolution of 3.6 Å. 3D classification was used to generate two 48-nm maps corresponding to different halves of the 96-nm repeat. Map quality of the DMT was further improved using focused refinement with 60 different cylindrical masks. Map quality of external complexes was improved using particle re-centering, 3D classification, mask-focused local refinement, and multi-body refinement. The final maps were merged into a single composite map. Maps and Fourier Shell Correlation (FSC) curves for each major axonemal complex are provided in Extended Data Fig. 7.

a

Complex	Particle number	Resolution (Å)	Modeling
96 nm DMT pos1 consensus	90,482	4.1	7UNG
96 nm DMT pos2 consensus	87,789	4.1	7UNG
DMT local refinement	90,482/87,789	3.3-3.8	7UNG
ODA-DC	118,977	3.8	7RRO
ODA core	13,430	10.0	7KZM, SW
RS1 base	27,216	4.4	7JTS
RS1 head	33,348	13.6	7JTK, 7DMP
RS2 base	51,323	4.3	7JU4
RS2 head	14,770	10.6	7JTK, 7DMP
RS3 base	46,957	5.1	DN, AF2
RS3 head	17,617	13.4	-
N-DRC baseplate	75,820	3.5	7JU4
N-DRC	15,459	11.7	AF2
IDA*a* base	44,499	4.4	DN
IDA*b* base	8,218	8.8	AF2
IDA*c* base	33,783	4.1	DN
IDA*e* base	40,827	4.4	DN
IDA*g/d* base	46,581	5.7	DN, AF2
IDA*g/d* motor domain	46,581	9.3	SW
IDA*f* and IDA*a* motor domain	19,609	12.7	SW, AF2

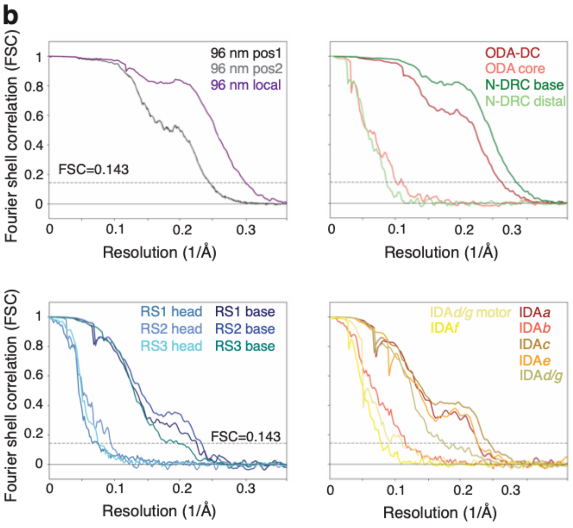

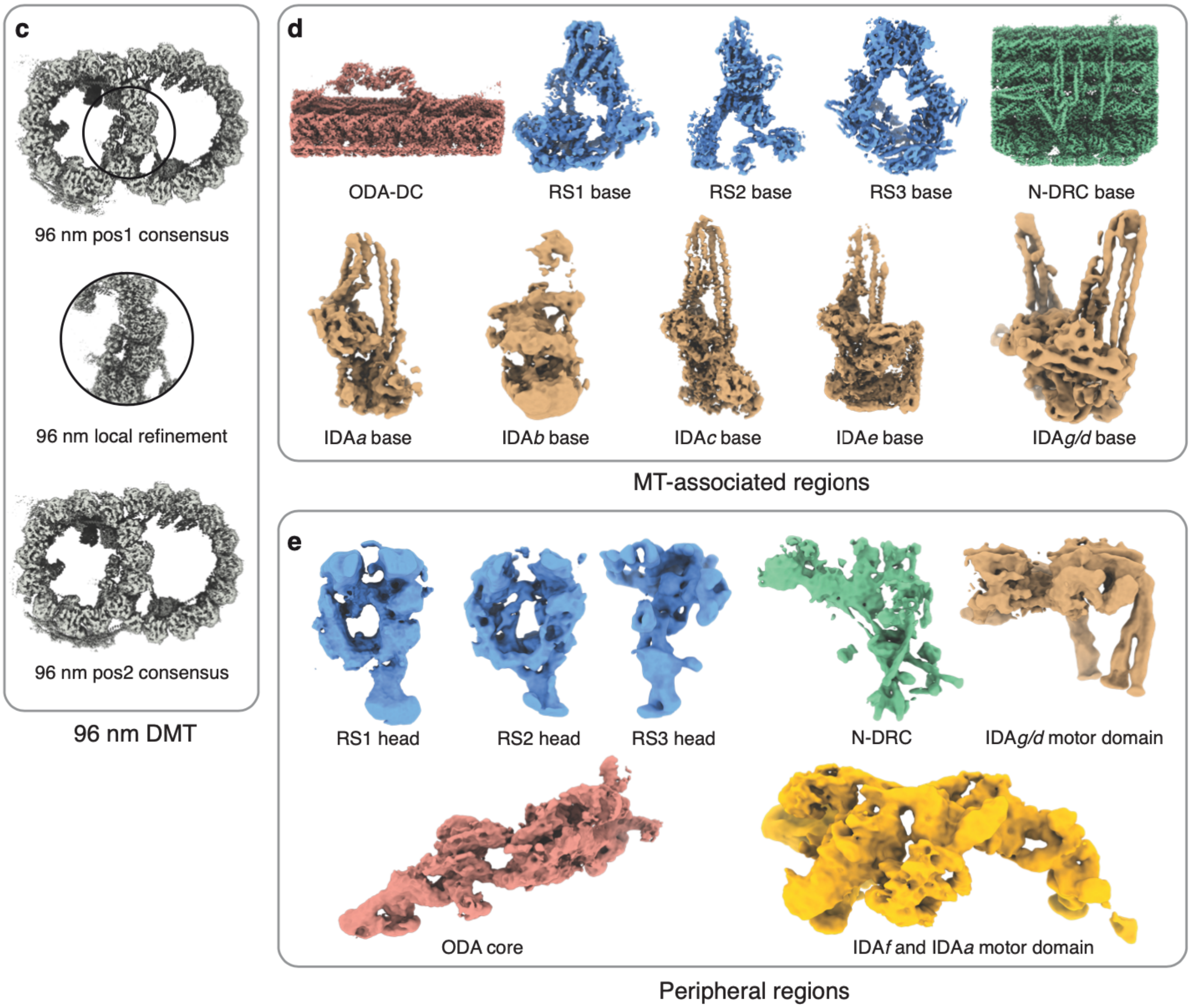

Supplementary Fig. 14 | Cryo-EM maps and Fourier Shell Correlation (FSC) curves for human axonemal complexes.
a, Summary table of the number of particles, achieved resolution, and modeling approach for each human axonemal complex. Abbreviations: DN, *de novo* model building; AF2, AlphaFold2-enabled model building; SW, homology

modeling using SWISS-MODEL. If a pre-existing PDB entry was used, its 4-character accession code is provided. **b**, FSC curves for the complexes listed in panel a. The resolution was determined using the FSC=0.143 criterion. **c**, Maps of the two different 48-nm sections (pos1 and pos2) that form the full 96-nm repeat. The circular inset exemplifies map improvement after local protofilament refinement. **d**, Maps of microtubule (MT)-associated regions following mask-focused local classification and refinement. **e**, Maps of peripheral regions following mask-focused local classification and refinement.

Section 5. SI video legends.

Supplementary Video 1. Rotation of two copies of the 96-nm repeat of the *C. reinhardtii* doublet microtubule. Complexes are colored according to the color scheme established in Fig. 1.

Supplementary Video 2. Coordinated movement of inner dynein arm (IDA) *f* with outer dynein arm (ODA) 1 and 2. Maps and models of *C. reinhardtii* ODA1, ODA2, and IDA*f* in closed and open states. To show the breaking of inter-ODA contacts, only the core regions and γ-HC motors of ODAs are shown. Contact with IDA*f* is maintained by ODA1 and ODA2, through the MIA complex, in both states. Open and closed states are highly correlated for ODA1 and ODA2 (Fig. 3a).

Supplementary Video 3. Coordinated movement of the nexin-dynein regulatory complex (N-DRC) with outer dynein arm (ODA) 3. Maps and models of *C. reinhardtii* N-DRC, inner dynein arm *g* (IDA*g*), and ODA3 in the closed and open states. The N-DRC linker is cropped in the first view to highlight the connection to IDA*g* and ODA3 through DRC3 and DRC7. The movements between closed and open states support the proposed role of the N-DRC as a mechano-sensor that regulates ODA and IDA function.

Supplementary Video 4. High-speed video microscopy of human respiratory cilia. Movies showing respiratory cilia from non-PCD controls, patient ID01 (*ODAD1* slice site mutation), patient ID02 (*ODAD1* nonsense mutation), patient ID03 (*CCDC40* mutation), and patient ID04 (*CCDC39* mutation).

SI References

85. Leung, M. *et al.* Unraveling the intricate microtubule inner protein networks that reinforce mammalian sperm flagella. (2022) doi:10.1101/2022.09.29.510157.

86. Mitchell, D. R. & Rosenbaum, J. L. Protein-protein interactions in the 18S ATPase of Chlamydomonas outer dynein arms. *Cell Motil. Cytoskeleton* 6, 510–520 (1986).

87. Sakakibara, H., Takada, S., King, S. M., Witman, G. B. & Kamiya, R. A Chlamydomonas outer arm dynein mutant with a truncated beta heavy chain. *J. Cell Biol.* 122, 653–661 (1993).

88. Mali, G. R. *et al.* Shulin packages axonemal outer dynein arms for ciliary targeting. *Science* 371, 910–916 (2021).

89. Sakakibara, H., Mitchell, D. R. & Kamiya, R. A Chlamydomonas outer arm dynein mutant missing the alpha heavy chain. *J. Cell Biol.* 113, 615–622 (1991).

90. Harrison, A. *et al.* Redox-based control of the gamma heavy chain ATPase from Chlamydomonas outer arm dynein. *Cell Motil. Cytoskeleton* 52, 131–143 (2002).

91. Kamiya, R., Kurimoto, E. & Muto, E. Two types of Chlamydomonas flagellar mutants missing different components of inner-arm dynein. *J. Cell Biol.* 112, 441–447 (1991).

92. Myster, S. H., Knott, J. A., Wysocki, K. M., O'Toole, E. & Porter, M. E. Domains in the 1alpha dynein heavy chain required for inner arm assembly and flagellar motility in Chlamydomonas. *J. Cell Biol.* 146, 801–818 (1999).

93. Perrone, C. A., Myster, S. H., Bower, R., O'Toole, E. T. & Porter, M. E. Insights into the structural organization of the I1 inner arm dynein from a domain analysis of the 1beta dynein heavy chain. *Mol. Biol. Cell* 11, 2297–2313 (2000).

94. Bower, R. *et al.* IC138 defines a subdomain at the base of the I1 dynein that regulates microtubule sliding and flagellar motility. *Mol. Biol. Cell* 20, 3055–3063 (2009).

95. Fu, G. *et al.* Structural organization of the intermediate and light chain complex of Chlamydomonas ciliary I1 dynein. *FASEB J.* 35, e21646 (2021).

96. Hendrickson, T. W. *et al.* IC138 is a WD-repeat dynein intermediate chain required for light chain assembly and regulation of flagellar bending. *Mol. Biol. Cell* 15, 5431–5442 (2004).

97. Perrone, C. A., Yang, P., O'Toole, E., Sale, W. S. & Porter, M. E. The Chlamydomonas IDA7 locus encodes a 140-kDa dynein intermediate chain required to assemble the I1 inner arm complex. *Mol. Biol. Cell* 9, 3351–3365 (1998).

98. Yang, P. & Sale, W. S. The Mr 140,000 intermediate chain of Chlamydomonas flagellar inner arm dynein is a WD-repeat protein implicated in dynein arm anchoring. *Mol. Biol. Cell* 9, 3335–3349 (1998).

99. DiBella, L. M., Sakato, M., Patel-King, R. S., Pazour, G. J. & King, S. M. The LC7 Light Chains of Chlamydomonas Flagellar Dyneins Interact with Components Required for Both Motor Assembly and Regulation. *Mol. Biol. Cell* 15, 4633–4646 (2004).

100. Harrison, A., Olds-Clarke, P. & King, S. M. Identification of the t complex-encoded cytoplasmic dynein light chain tctex1 in inner arm I1 supports the involvement of flagellar dyneins in meiotic drive. *J. Cell Biol.* 140, 1137–1147 (1998).

101. Toropova, K. *et al.* Structure of the dynein-2 complex and its assembly with intraflagellar transport trains. *Nat. Struct. Mol. Biol.* 26, 823–829 (2019).

102. DiBella, L. M., Smith, E. F., Patel-King, R. S., Wakabayashi, K. & King, S. M. A novel Tctex2-related light chain is required for stability of inner dynein arm I1 and motor function in the Chlamydomonas flagellum. *J. Biol. Chem.* 279, 21666–21676 (2004).

103. Beneke, T., Banecki, K., Fochler, S. & Gluenz, E. LAX28 is required for the stable assembly of the inner dynein arm f complex, and the tether and tether head complex in Leishmania flagella. *J. Cell. Sci.* 133, (2020).

104. King, S. J., Inwood, W. B., O'Toole, E. T., Power, J. & Dutcher, S. K. The bop2-1 mutation reveals radial asymmetry in the inner dynein arm region of Chlamydomonas reinhardtii. *J. Cell Biol.* 126, 1255–1266 (1994).

105. Bustamante-Marin, X. M. *et al.* Mutation of CFAP57, a protein required for the asymmetric targeting of a subset of inner dynein arms in Chlamydomonas, causes primary ciliary dyskinesia. *Plos Genet* 16, e1008691 (2020).

106. He, X. *et al.* Bi-allelic Loss-of-function Variants in CFAP58 Cause Flagellar Axoneme and Mitochondrial Sheath Defects and Asthenoteratozoospermia in Humans and Mice. *Am. J. Hum. Genet.* 107, 514–526 (2020).

107. Song, K. *et al.* In situ localization of N and C termini of subunits of the flagellar nexin-dynein regulatory complex (N-DRC) using SNAP tag and cryo-electron tomography. *J. Biol. Chem.* 290, 5341–5353 (2015).

108. Lin, J. *et al.* Building blocks of the nexin-dynein regulatory complex in Chlamydomonas flagella. *J. Biol. Chem.* 286, 29175–29191 (2011).

109. Bower, R. *et al.* The N-DRC forms a conserved biochemical complex that maintains outer doublet alignment and limits microtubule sliding in motile axonemes. *Mol. Biol. Cell* 24, 1134–1152 (2013).

110. Greenan, G. A., Vale, R. D. & Agard, D. A. Electron cryotomography of intact motile cilia defines the basal body to axoneme transition. *J. Cell Biol.* 219, 241 (2020).

111. Urbanska, P. *et al.* The CSC proteins FAP61 and FAP251 build the basal substructures of radial spoke 3 in cilia. *Mol. Biol. Cell* 26, 1463–1475 (2015).

112. LeDizet, M. & Piperno, G. The light chain p28 associates with a subset of inner dynein arm heavy chains in Chlamydomonas axonemes. *Mol. Biol. Cell* 6, 697–711 (1995).

113. Yanagisawa, H. & Kamiya, R. Association between Actin and Light Chains in Chlamydomonas Flagellar Inner-Arm Dyneins. *Biochemical and Biophysical Research Communications* 288, 443–447 (2001).

114. Yamamoto, R., Yanagisawa, H., Yagi, T. & Kamiya, R. A novel subunit of axonemal dynein conserved among lower and higher eukaryotes. *Febs Lett* 580, 6357–6360 (2006).

115. Yamamoto, R., Yanagisawa, H., Yagi, T. & Kamiya, R. Novel 44-kilodalton subunit of axonemal Dynein conserved from chlamydomonas to mammals. *Eukaryotic Cell* 7, 154–161 (2008).

116. Zhang, X. *et al.* LRRC23 is a conserved component of the radial spoke that is necessary for sperm motility and male fertility in mice. *J Cell Sci* 134, (2021).

117. Loges, N. T. *et al.* Recessive DNAH9 Loss-of-Function Mutations Cause Laterality Defects and Subtle Respiratory Ciliary-Beating Defects. *Am J Hum Genet* 103, 995–1008 (2018).

118. Keays, D. A. *et al.* Mutations in α-Tubulin Cause Abnormal Neuronal Migration in Mice and Lissencephaly in Humans. *Cell* 128, 45–57 (2007).

119. Mechaussier, S. *et al.* TUBB4B variants specifically impact ciliary function, causing a ciliopathic spectrum. *medRxiv* 2022.10.19.22280748 (2022) doi:10.1101/2022.10.19.22280748.

120. Luscan, R. *et al.* Mutations in TUBB4B Cause a Distinctive Sensorineural Disease. *Am J Hum Genetics* 101, 1006–1012 (2017).

121. Pozo, M. G. *et al.* A comprehensive WGS-based pipeline for the identification of new candidate genes in inherited retinal dystrophies. *Npj Genom Medicine* 7, 17 (2022).

122. Dougherty, G. W. *et al.* CFAP45 deficiency causes situs abnormalities and asthenospermia by disrupting an axonemal adenine nucleotide homeostasis module. *Nat Commun* 11, 5520–20 (2020).

123. Ta-Shma, A. *et al.* A human laterality disorder associated with a homozygous WDR16 deletion. *Eur. J. Hum. Genet.* 23, 1262–1265 (2015).

124. Narasimhan, V. *et al.* Mutations in CCDC11, which encodes a coiled-coil containing ciliary protein, causes situs inversus due to dysmotility of monocilia in the left-right organizer. *Hum. Mutat.* 36, 307–318 (2015).

125. Perles, Z. *et al.* A human laterality disorder associated with recessive CCDC11 mutation. *J. Med. Genet.* 49, 386–390 (2012).

126. Noël, E. S. *et al.* A Zebrafish Loss-of-Function Model for Human CFAP53 Mutations Reveals Its Specific Role in Laterality Organ Function. *Hum. Mutat.* 37, 194–200 (2016).

127. Sun, S.-C. *et al.* Mutations in C1orf194, encoding a calcium regulator, cause dominant Charcot-Marie-Tooth disease. *Brain* 142, 2215–2229 (2019).

128. Gonsales, M. C. *et al.* Revisiting the clinical impact of variants in EFHC1 in patients with different phenotypes of genetic generalized epilepsy. *Epilepsy Behav* 112, 107469 (2020).

129. Gu, W., Sander, T., Heils, A., Lenzen, K. P. & Steinlein, O. K. A new EF-hand containing gene EFHC2 on Xp11.4: Tentative evidence for association with juvenile myoclonic epilepsy. *Epilepsy Res* 66, 91–98 (2005).

130. Sigg, M. A. *et al.* Evolutionary Proteomics Uncovers Ancient Associations of Cilia with Signaling Pathways. *Dev Cell* 43, 744-762.e11 (2017).

131. Shamseldin, H. E. *et al.* The morbid genome of ciliopathies: an update. *Genet Med* 22, 1051–1060 (2020).

132. Ta-Shma, A. *et al.* Homozygous loss-of-function mutations in MNS1 cause laterality defects and likely male infertility. *Plos Genet* 14, e1007602 (2018).

133. Leslie, J. S. *et al.* MNS1 variant associated with situs inversus and male infertility. *Eur J Hum Genet* 28, 50–55 (2020).

134. Li, Y. *et al.* A novel homozygous frameshift mutation in MNS1 associated with severe oligoasthenoteratozoospermia in humans. *Asian J Androl* 23, 197–204 (2020).

135. Reish, O. *et al.* A Homozygous Nme7 Mutation Is Associated with Situs Inversus Totalis. *Hum Mutat* 37, 727–31 (2015).

136. Mira, M. T. *et al.* Susceptibility to leprosy is associated with PARK2 and PACRG. *Nature* 427, 636–640 (2004).

137. Ryan, R. *et al.* Functional characterization of tektin-1 in motile cilia and evidence for TEKT1 as a new candidate gene for motile ciliopathies. *Hum Mol Genet* 27, 266–282 (2018).

138. Antony, D. *et al.* Mutations in CCDC39 and CCDC40 are the major cause of primary ciliary dyskinesia with axonemal disorganization and absent inner dynein arms. *Hum. Mutat.* 34, 462–472 (2013).

139. Merveille, A.-C. *et al.* CCDC39 is required for assembly of inner dynein arms and the dynein regulatory complex and for normal ciliary motility in humans and dogs. *Nat. Genet.* 43, 72–78 (2011).

140. Becker-Heck, A. *et al.* The coiled-coil domain containing protein CCDC40 is essential for motile cilia function and left-right axis formation. *Nat. Genet.* 43, 79–84 (2011).

141. Sha, Y. *et al.* Biallelic mutations of CFAP58 are associated with multiple morphological abnormalities of the sperm flagella. *Clin. Genet.* 99, 443–448 (2021).

142. Oud, M. S. *et al.* Exome sequencing reveals variants in known and novel candidate genes for severe sperm motility disorders. *Hum Reprod* 36, 2597–2611 (2021).

143. Khouri, E. E. *et al.* Mutations in DNAJB13, Encoding an HSP40 Family Member, Cause Primary Ciliary Dyskinesia and Male Infertility. *Am. J. Hum. Genet.* 99, 489–500 (2016).

144. Cho, E. H. *et al.* A nonsense variant in NME5 causes human primary ciliary dyskinesia with radial spoke defects. *Clin Genet* 98, 64–68 (2020).

145. Cho, E. H. *et al.* Genetic Analysis of Korean Adult Patients with Nontuberculous Mycobacteria Suspected of Primary Ciliary Dyskinesia Using Whole Exome Sequencing. *Yonsei Med J* 62, 224–230 (2021).

146. Kott, E. *et al.* Loss-of-function mutations in RSPH1 cause primary ciliary dyskinesia with central-complex and radial-spoke defects. *Am. J. Hum. Genet.* 93, 561–570 (2013).

147. Onoufriadis, A. *et al.* Targeted NGS gene panel identifies mutations in RSPH1 causing primary ciliary dyskinesia and a common mechanism for ciliary central pair agenesis due to radial spoke defects. *Hum. Mol. Genet.* 23, 3362–3374 (2014).

148. Jeanson, L. *et al.* RSPH3 Mutations Cause Primary Ciliary Dyskinesia with Central-Complex Defects and a Near Absence of Radial Spokes. *Am. J. Hum. Genet.* 97, 153–162 (2015).

149. Castleman, V. H. *et al.* Mutations in Radial Spoke Head Protein Genes RSPH9 and RSPH4A Cause Primary Ciliary Dyskinesia with Central-Microtubular-Pair Abnormalities. *Am J Hum Genetics* 84, 197–209 (2009).

150. Daniels, M. L. A. *et al.* Founder Mutation in RSPH4A Identified in Patients of Hispanic Descent with Primary Ciliary Dyskinesia. *Hum Mutat* 34, 1352–1356 (2013).

151. Wang, L. *et al.* Novel RSPH4A Variants Associated With Primary Ciliary Dyskinesia–Related Infertility in Three Chinese Families. *Frontiers Genetics* 13, 922287 (2022).

152. Liu, S. *et al.* CFAP61 is required for sperm flagellum formation and male fertility in human and mouse. *Development* 148,.

153. Hu, T. *et al.* Biallelic CFAP61 variants cause male infertility in humans and mice with severe oligoasthenoteratozoospermia. *J Med Genet* jmedgenet-2021-108249 (2022) doi:10.1136/jmedgenet-2021-108249.

154. Ma, A. *et al.* Biallelic Variants in CFAP61 Cause Multiple Morphological Abnormalities of the Flagella and Male Infertility. *Frontiers Cell Dev Biology* 9, 803818 (2022).

155. Martinez, G. *et al.* Biallelic variants in MAATS1 encoding CFAP91, a calmodulin-associated and spoke-associated complex protein, cause severe astheno-teratozoospermia and male infertility. *J Med Genet* 57, 708–716 (2020).

156. Auguste, Y. *et al.* Loss of Calmodulin- and Radial-Spoke-Associated Complex Protein CFAP251 Leads to Immotile Spermatozoa Lacking Mitochondria and Infertility in Men. *Am J Hum Genetics* 103, 413–420 (2018).

157. Li, W. *et al.* Biallelic mutations of CFAP251 cause sperm flagellar defects and human male infertility. *J Hum Genet* 64, 49–54 (2019).

158. Kherraf, Z.-E. *et al.* A Homozygous Ancestral SVA-Insertion-Mediated Deletion in WDR66 Induces Multiple Morphological Abnormalities of the Sperm Flagellum and Male Infertility. *Am J Hum Genetics* 103, 400–412 (2018).

159. Wirschell, M. *et al.* The nexin-dynein regulatory complex subunit DRC1 is essential for motile cilia function in algae and humans. *Nat Genet* 45, 262–268 (2013).

160. Raidt, J. *et al.* Ciliary beat pattern and frequency in genetic variants of primary ciliary dyskinesia. *Eur Respir J* 44, 1579–1588 (2014).

161. Lei, C. *et al.* DRC1 deficiency caused primary ciliary dyskinesia and MMAF in a Chinese patient. *J Hum Genet* 67, 197–201 (2022).

162. Zhang, J. *et al.* Loss of DRC1 function leads to multiple morphological abnormalities of the sperm flagella and male infertility in human and mouse. *Hum Mol Genet* 30, 1996–2011 (2021).

163. Horani, A. *et al.* CCDC65 Mutation Causes Primary Ciliary Dyskinesia with Normal Ultrastructure and Hyperkinetic Cilia. *Plos One* 8, e72299 (2013).

164. Austin-Tse, C. *et al.* Zebrafish Ciliopathy Screen Plus Human Mutational Analysis Identifies C21orf59 and CCDC65 Defects as Causing Primary Ciliary Dyskinesia. *Am J Hum Genetics* 93, 672–686 (2013).

165. Jeanson, L. *et al.* Mutations in GAS8, a Gene Encoding a Nexin-Dynein Regulatory Complex Subunit, Cause Primary Ciliary Dyskinesia with Axonemal Disorganization. *Hum Mutat* 37, 776–785 (2016).

166. Olbrich, H. *et al.* Loss-of-Function GAS8 Mutations Cause Primary Ciliary Dyskinesia and Disrupt the Nexin-Dynein Regulatory Complex. *Am J Hum Genetics* 97, 546–554 (2015).

167. Lewis, W. R. *et al.* Mutation of Growth Arrest Specific 8 Reveals a Role in Motile Cilia Function and Human Disease. *Plos Genet* 12, e1006220 (2016).

168. Zhou, S. *et al.* Bi-allelic variants in human TCTE1/DRC5 cause asthenospermia and male infertility. *Eur J Hum Genet* 30, 721–729 (2022).

169. Imtiaz, F., Allam, R., Ramzan, K. & Al-Sayed, M. Variation in DNAH1 may contribute to primary ciliary dyskinesia. *Bmc Med Genet* 16, 14 (2015).

170. Ben Khelifa, M. *et al.* Mutations in DNAH1, which Encodes an Inner Arm Heavy Chain Dynein, Lead to Male Infertility from Multiple Morphological Abnormalities of the Sperm Flagella. *Am J Hum Genetics* 94, 95–104 (2014).

171. Li, Y. *et al.* DNAH6 and Its Interactions with PCD Genes in Heterotaxy and Primary Ciliary Dyskinesia. *Plos Genet* 12, e1005821 (2016).

172. Tu, C. *et al.* Identification of DNAH6 mutations in infertile men with multiple morphological abnormalities of the sperm flagella. *Sci Rep-uk* 9, 15864 (2019).

173. Gershoni, M. *et al.* A familial study of azoospermic men identifies three novel causative mutations in three new human azoospermia genes. *Genet Med* 19, 998–1006 (2017).

174. Zhang, Y. J. *et al.* Identification of Dynein Heavy Chain 7 as an Inner Arm Component of Human Cilia That Is Synthesized but Not Assembled in a Case of Primary Ciliary Dyskinesia*. *J Biol Chem* 277, 17906–17915 (2002).

175. Wei, X. *et al.* Bi-allelic mutations in DNAH7 cause asthenozoospermia by impairing the integrality of axoneme structure. *Acta Bioch Bioph Sin* 53, 1300–1309 (2021).

176. Gao, Y. *et al.* Loss of function mutation in DNAH7 induces male infertility associated with abnormalities of the sperm flagella and mitochondria in human. *Clin Genet* 102, 130–135 (2022).

177. Li, Y. *et al.* Whole-exome sequencing of a cohort of infertile men reveals novel causative genes in teratozoospermia that are chiefly related to sperm head defects. *Hum Reprod* 37, 152–177 (2021).

178. Liu, C. *et al.* Bi-allelic Mutations in TTC29 Cause Male Subfertility with Asthenoteratospermia in Humans and Mice. *Am J Hum Genetics* 105, 1168–1181 (2019).

179. Lorès, P. *et al.* Mutations in TTC29, Encoding an Evolutionarily Conserved Axonemal Protein, Result in Asthenozoospermia and Male Infertility. *Am J Hum Genetics* 105, 1148–1167 (2019).

180. Son, J.-H. *et al.* A genome-wide association study on frequent exacerbation of asthma depending on smoking status. *Resp Med* 199, 106877 (2022).

181. Li, Y. *et al.* DNAH2 is a novel candidate gene associated with multiple morphological abnormalities of the sperm flagella. *Clin Genet* 95, 590–600 (2019).

182. Gao, Y. *et al.* Novel bi-allelic variants in DNAH2 cause severe asthenoteratozoospermia with multiple morphological abnormalities of the flagella. *Reprod Biomed Online* 42, 963–972 (2021).

183. Hwang, J. Y. *et al.* Genetic Defects in DNAH2 Underlie Male Infertility With Multiple Morphological Abnormalities of the Sperm Flagella in Humans and Mice. *Frontiers Cell Dev Biology* 9, 662903 (2021).

184. Tu, C. *et al.* Bi-allelic mutations of DNAH10 cause primary male infertility with asthenoteratozoospermia in humans and mice. *Am J Hum Genetics* 108, 1466–1477 (2021).

185. Li, K. *et al.* Bi-allelic variants in DNAH10 cause asthenoteratozoospermia and male infertility. *J Assist Reprod Gen* 39, 251–259 (2022).

186. Lu, S. *et al.* Bi-allelic variants in human WDR63 cause male infertility via abnormal inner dynein arms assembly. *Cell Discov* 7, 110 (2021).

187. Zhang, Z. *et al.* Positional cloning of the major quantitative trait locus underlying lung tumor susceptibility in mice. *Proc National Acad Sci* 100, 12642–12647 (2003).

188. Schmidts, M. *et al.* TCTEX1D2 mutations underlie Jeune asphyxiating thoracic dystrophy with impaired retrograde intraflagellar transport. *Nat Commun* 6, 7074 (2015).

189. Tessema, M. *et al.* Epigenetic Repression of CCDC37 and MAP1B Links Chronic Obstructive Pulmonary Disease to Lung Cancer. *J Thorac Oncol* 10, 1181–1188 (2015).

190. Rorick, N. K. *et al.* Genomic strategy identifies a missense mutation in WD-repeat domain 65 (WDR65) in an individual with Van der Woude syndrome. *Am J Medical Genetics Part* 155A, 1314–21 (2010).

191. Tang, S. *et al.* Biallelic Mutations in CFAP43 and CFAP44 Cause Male Infertility with Multiple Morphological Abnormalities of the Sperm Flagella. *Am J Hum Genetics* 100, 854–864 (2017).

192. Morimoto, Y. *et al.* Nonsense mutation in CFAP43 causes normal-pressure hydrocephalus with ciliary abnormalities. *Neurology* 92, e2364–e2374 (2019).

193. Coutton, C. *et al.* Mutations in CFAP43 and CFAP44 cause male infertility and flagellum defects in Trypanosoma and human. *Nat Commun* 9, 686 (2018).

194. Fassad, M. R. *et al.* Mutations in Outer Dynein Arm Heavy Chain DNAH9 Cause Motile Cilia Defects and Situs Inversus. *Am J Hum Genetics* 103, 984–994 (2018).

195. Omran, H. *et al.* Homozygosity Mapping of a Gene Locus for Primary Ciliary Dyskinesia on Chromosome 5p and Identification of the Heavy Dynein Chain DNAH5 as a Candidate Gene. *Am J Resp Cell Mol* 23, 696–702 (2000).

196. Olbrich, H. *et al.* Mutations in DNAH5 cause primary ciliary dyskinesia and randomization of left-right asymmetry. *Nat Genet* 30, 143–4 (2002).

197. Guichard, C. *et al.* Axonemal Dynein Intermediate-Chain Gene (DNAI1) Mutations Result in Situs Inversus and Primary Ciliary Dyskinesia (Kartagener Syndrome). *Am J Hum Genetics* 68, 1030–1035 (2001).

198. Loges, N. T. *et al.* DNAI2 Mutations Cause Primary Ciliary Dyskinesia with Defects in the Outer Dynein Arm. *Am J Hum Genetics* 83, 547–558 (2008).

199. Mazor, M. *et al.* Primary Ciliary Dyskinesia Caused by Homozygous Mutation in DNAL1, Encoding Dynein Light Chain 1. *Am J Hum Genetics* 88, 599–607 (2011).

200. Knowles, M. R. *et al.* Exome Sequencing Identifies Mutations in CCDC114 as a Cause of Primary Ciliary Dyskinesia. *Am J Hum Genetics* 92, 99–106 (2013).

201. Onoufriadis, A. *et al.* Combined exome and whole-genome sequencing identifies mutations in ARMC4 as a cause of primary ciliary dyskinesia with defects in the outer dynein arm. *J Med Genet* 51, 61–67 (2014).

202. Hjeij, R. *et al.* ARMC4 Mutations Cause Primary Ciliary Dyskinesia with Randomization of Left/Right Body Asymmetry. *Am J Hum Genetics* 93, 357–367 (2013).

203. Alsaadi, M. M. *et al.* Nonsense Mutation in Coiled-Coil Domain Containing 151 Gene (CCDC151) Causes Primary Ciliary Dyskinesia. *Hum Mutat* 35, 1446–1448 (2014).

204. Hjeij, R. *et al.* CCDC151 Mutations Cause Primary Ciliary Dyskinesia by Disruption of the Outer Dynein Arm Docking Complex Formation. *Am J Hum Genetics* 95, 257–274 (2014).

205. Wallmeier, J. *et al.* TTC25 Deficiency Results in Defects of the Outer Dynein Arm Docking Machinery and Primary Ciliary Dyskinesia with Left-Right Body Asymmetry Randomization. *Am J Hum Genetics* 99, 460–469 (2016).

206. Hjeij, R. *et al.* Pathogenic variants in CLXN encoding the Outer Dynein Arm Docking associated calcium-binding protein calaxin cause primary ciliary dyskinesia. *Genet Med* 100798 (2023) doi:10.1016/j.gim.2023.100798.
