# Supplementary material for: Axonemal structures reveal mechanoregulatory and disease mechanisms
Source: Nature. 2023 May 31;618(7965):625–33. doi: 10.1038/s41586-023-06140-2 (PMC10266980; doi:10.1038/s41586-023-06140-2)
Supplement: Supplementary file 2 — Reporting Summary [file 41586_2023_6140_MOESM2_ESM.pdf]

nature portfolio

Corresponding author(s): Alan Brown

Last updated by author(s): Apr 10, 2023

Reporting Summary

Nature Portfolio wishes to improve the reproducibility of the work that we publish. This form provides structure for consistency and transparency in reporting. For further information on Nature Portfolio policies, see our Editorial Policies and the Editorial Policy Checklist.

Statistics

For all statistical analyses, confirm that the following items are present in the figure legend, table legend, main text, or Methods section.

n/a	Confirmed	
☐	☒	The exact sample size (*n*) for each experimental group/condition, given as a discrete number and unit of measurement
☐	☒	A statement on whether measurements were taken from distinct samples or whether the same sample was measured repeatedly
☐	☒	The statistical test(s) used AND whether they are one- or two-sided<br>*Only common tests should be described solely by name; describe more complex techniques in the Methods section.*
☒	☐	A description of all covariates tested
☒	☐	A description of any assumptions or corrections, such as tests of normality and adjustment for multiple comparisons
☐	☒	A full description of the statistical parameters including central tendency (e.g. means) or other basic estimates (e.g. regression coefficient) AND variation (e.g. standard deviation) or associated estimates of uncertainty (e.g. confidence intervals)
☒	☐	For null hypothesis testing, the test statistic (e.g. *F*, *t*, *r*) with confidence intervals, effect sizes, degrees of freedom and *P* value noted<br>*Give P values as exact values whenever suitable.*
☒	☐	For Bayesian analysis, information on the choice of priors and Markov chain Monte Carlo settings
☒	☐	For hierarchical and complex designs, identification of the appropriate level for tests and full reporting of outcomes
☒	☐	Estimates of effect sizes (e.g. Cohen's *d*, Pearson's *r*), indicating how they were calculated

Our web collection on statistics for biologists contains articles on many of the points above.

Software and code

Policy information about availability of computer code

Data collection	SerialEM v3.6 and v3.7
Data analysis	RELION-3.1; RELION-4.0; Chimera v1.15; ChimeraX v1.3; Coot v0.9.4.1; phenix.real_space_refine v1.19.2-4158; Phenix.molprobity v1.19.2-4158; SWISS-MODEL; AlphaFold v2.1.1; AlphaFold v2.2.0; R v4.0.3; MotionCor2; CTFFIND4; Namdinator (no version number); PDBeFOLD v2.59; Clustal Omega Web Service (no version number); DeepTracer Web Service (no version number); Matlab R2022a; GraphPad Prism v. 8.0.0, Zenodo (https://doi.org/10.5281/zenodo.6908250).

For manuscripts utilizing custom algorithms or software that are central to the research but not yet described in published literature, software must be made available to editors and reviewers. We strongly encourage code deposition in a community repository (e.g. GitHub). See the Nature Portfolio guidelines for submitting code & software for further information.

Data

Policy information about availability of data

All manuscripts must include a data availability statement. This statement should provide the following information, where applicable:

- Accession codes, unique identifiers, or web links for publicly available datasets
- A description of any restrictions on data availability
- For clinical datasets or third party data, please ensure that the statement adheres to our policy

A composite cryo-EM map of the 96-nm repeat unit of DMTs from C. reinhardtii flagella has been deposited to the Electron Microscopy Data Bank (EMDB; https://

www.ebi.ac.uk/pdbe/emdb/) with the accession code EMD-40220. The atomic model of the C. reinhardtii DMT has been deposited in the Protein Data Bank (PDB; https://www.rcsb.org/) with accession code 8GLV. A composite cryo-EM map of the 96-nm repeat unit of DMTs from human respiratory cilia has been deposited in the EMDB with accession code EMD-35888. The atomic model of the human DMT has been deposited in the PDB with accession code 8J07. For both EMD-40220 and EMD-35888, half maps of the individual axonemal complexes and the masks used for focused refinement have been deposited as additional maps associated with the entries. Data and code used to calculate the correlation between open and closed states of the ODA is available at https://doi.org/10.5281/zenodo.6908250.

Human research participants

Policy information about studies involving human research participants and Sex and Gender in Research.

Reporting on sex and gender	Sex or gender was not considered in the study design. All human cilia samples were obtained from males.
Population characteristics	Nasal brushings were collected from four individuals with PCD. These individuals were aged 9, 17, 22, and 40. All individuals were determined to have PCD using standard diagnostic tests. All had recurrent chest infections, continuous wet productive coughs and persistent rhinorrhoea.
Recruitment	The samples with genotypes of interest were obtained retrospectively from patients recruited as part of ongoing genetic research projects.
Ethics oversight	Peripheral blood samples for genetic screening, and respiratory epithelial cells collected from the inferior nasal turbinate by nasal scrape biopsy, were obtained from affected individuals and healthy volunteers under ethical approval granted through the Health Research Authority London Bloomsbury Research Ethics Committee (REC 08/H0713/82; IRAS 103488) and Living Airway Biobank, administered through the UCL Great Ormond Street Institute of Child Health (REC 19/NW/0171, IRAS 261511, Health Research Authority North West Liverpool East Research Ethics Committee). Informed written consent was obtained from all participants prior to enrollment in the study.

Note that full information on the approval of the study protocol must also be provided in the manuscript.

Field-specific reporting

Please select the one below that is the best fit for your research. If you are not sure, read the appropriate sections before making your selection.

☒ Life sciences ☐ Behavioural & social sciences ☐ Ecological, evolutionary & environmental sciences

For a reference copy of the document with all sections, see nature.com/documents/nr-reporting-summary-flat.pdf

Life sciences study design

All studies must disclose on these points even when the disclosure is negative.

Sample size	1. For cilia beat analysis, movie recordings from 11 non-PCD controls and 4 PCD patients were used. Number of movies = 104 (control); 3 (ODAD1 splice); 8 (ODAD1 nonsense); 8 (CCDC40), and 6 (CCDC39). No methods were used to predetermine sample size.<br>2. For cryo-EM processing, no methods were used to predetermine sample size. Final structures of the C. reinhardtii and H. sapiens 96-nm repeat were calculated from a total of 31,275 and 16,933 micrographs, respectively. The final number of micrographs was determined to be sufficient as they provided structures of doublet microtubules to better than 4 Angstrom resolution.<br>3. Sample size for odds ratio calculations were determined by unsupervised 3D classification using RELION software to generate 160,444 particles for ODA1, 162,179 particles for ODA2, 214,203 particles for ODA3, and 177,762 particles for ODA4.
Data exclusions	1. For the cilia beat analysis, static cilia were excluded from the analysis.<br>2. The algorithms used for single particle image processing may down-weight or exclude particles as part of their refinement strategy.
Replication	1. Multiple cryo-EM datasets were collected for the wild-type doublet microtubule structures. Their reconstructions were consistent, and merged.<br>2. For the ciliary beat analysis, between 3 and 104 different movies were analyzed (see Sample Size above).
Randomization	For calculation of the Fourier Shell Correlation (FSC), cryo-EM particles were randomly split into two halves using the RELION software.
Blinding	1. The initial diagnostic assessment was blinded, as these tests were done before a confirmed genetic result. As up to 80% of patients referred for diagnostic testing are found not to have PCD, there should not be any bias to influence these results.<br>2. Blinding was not used for the cilia beat analysis. However, the methods are objective and should not introduce bias or impact the measurements.<br>3. Blinding was not used for the cryo-EM studies; the researchers were aware of the identity of the samples. However, similar processing steps were used for the wild-type and PCD samples.

Reporting for specific materials, systems and methods

We require information from authors about some types of materials, experimental systems and methods used in many studies. Here, indicate whether each material, system or method listed is relevant to your study. If you are not sure if a list item applies to your research, read the appropriate section before selecting a response.

Materials & experimental systems

n/a	Involved in the study
☒	☐ Antibodies
☒	☐ Eukaryotic cell lines
☒	☐ Palaeontology and archaeology
☒	☐ Animals and other organisms
☒	☐ Clinical data
☒	☐ Dual use research of concern

Methods

n/a	Involved in the study
☒	☐ ChIP-seq
☒	☐ Flow cytometry
☒	☐ MRI-based neuroimaging
